# Dual-screw versus single-screw cephalomedullary nails for intertrochanteric femoral fractures: a systematic review and meta-analysis

**DOI:** 10.1186/s13018-023-04103-x

**Published:** 2023-08-20

**Authors:** Fan Yang, Xiafei Li, Lei Zhao, Qi Yang

**Affiliations:** Department of Traumatic Orthopedics, Yuncheng Central Hospital, Shanxi Medical University, No. 3690, Hedong East Street, Yanhu District, Yuncheng City, 044000 Shanxi Province China

**Keywords:** Intertrochanteric femoral fractures, InterTAN, Proximal femoral nail anti-rotation, Gamma3 nail

## Abstract

**Background:**

Internal fixation with cephalomedullary nails has been widely used in the treatment of intertrochanteric femoral fractures (IFF). Yet, the difference in efficacy and safety between the commonly used integrated dual-screw cephalomedullary nail (InterTAN) and single-screw cephalomedullary nail remains inconclusive. Thus we performed the present systematic review and meta-analysis.

**Methods:**

Randomized controlled trials (RCTs) or observational studies comparing InterTAN with proximal femoral nail anti-rotation (PFNA), the Asian PFNA (PFNA-II), or the Gamma3 nail in treating IFF were searched on PubMed, EMBASE, Web of Science and Cochrane Library from inception to April 30, 2023. The differences in perioperative parameters and clinical and radiological outcomes were evaluated by mean difference (MD) with 95% confidence interval (95%CI). The risks of various complications and mortality were assessed by risk ratio (RR) with 95%CI.

**Results:**

Twenty-three studies comprising 3566 patients were included. Compared with single-screw cephalomedullary nails (PFNA/PFNA-II, Gamma3), InterTAN conferred significantly reduced risk of implant failures (RR = 0.37, 95%CI 0.26 to 0.51, *P* < 0.001), hip and thigh pain (RR = 0.70, 95%CI 0.55 to 0.90, *P* = 0.006) and all-cause revision/reoperation (RR = 0.38, 95%CI 0.26 to 0.57, *P* < 0.001). Moreover, patients treated with InterTAN had significantly higher 1-year Harris Hip Score (MD = 0.82, 95%CI 0.20–1.44, *P* = 0.010) and shorter time to union/healing (MD = − 0.66 days, 95%CI  − 1.16 to  − 0.16, *P* = 0.009). Femoral neck shortening, time to full bearing, and incidences of non-union, infection, deep venous thrombosis, and mortality were comparable between both groups.

**Conclusions:**

The integrated dual-screw InterTAN construct has superior performance in reducing risks of complications and improving clinical and functional outcomes in the treatment of IFF. More well-designed, high-quality RCTs are warranted to confirm these findings.

**Supplementary Information:**

The online version contains supplementary material available at 10.1186/s13018-023-04103-x.

## Background

Hip fractures are the major cause of morbidity, disability, and mortality among the elderly [1, 2]. Intertrochanteric femoral fractures (IFFs) are the most common type of hip fracture, which contribute to nearly half of all hip fractures [3]. As the aging population increases rapidly, the incidence of IFF is increasing, and the disease, economic and social burden caused by IFF becomes more and more heavy [4]. IFF has become a major global issue nowadays, especially among the elderly. Timely surgical treatment is preferred for IFF to reduce the risk of complications [5–7]. There are two major choices of internal fixations for IFF, extramedullary devices such as the dynamic hip screw (DHS) and cephalomedullary nails such as proximal femoral nail anti-rotation (PFNA) and InterTAN [8, 9]. Yet, cephalomedullary nails have biomechanical advantages over extramedullary devices, because they are closer to the force vector line and have shorter moment arms [10]. Cephalomedullary nails offer greater biomechanical stabilization, increase load bearing, reduce the risk of fixation failure, and lead to superior radiographic outcomes [11, 12]. Therefore, cephalomedullary devices are preferable to extramedullary devices and are more and more widely used, especially in unstable IFF according to the Arbeitsge-meinschaft für Osteosynthesefragen/Orthopaedic Trauma Association classification (AO/OTA 31A2 and A3) [13, 14].

There are several types of commonly used cephalomedullary nail systems, including proximal femoral nail anti-rotation (PFNA) or the Asian PFNA (PFNA-II), Gamma3 nail, and intertrochanteric antegrade nail (InterTAN). PFNA/PFNA-II and Gamma3 nails belong to single-screw cephalomedullary implants, whereas InterTAN uses two cephalomedullary screws. The design of the helical blade of PFNA helps compress the cancellous bone and increase the contact area with the bone to achieve tighter bone compaction and femur alignment [15, 16]. InterTAN offers inter-fragmentary compression with locking using an integrated dual-screw construct [17]. These cephalomedullary nails are minimally invasive, provide greater mechanical strength and anti-rotation stability, and result in fewer complications.

There are differences in mechanical performance between dual-screw and single-screw cephalomedullary nails. Biomechanical studies using cadaveric models demonstrate the dual-screw InterTAN system has better anti-rotation stability and greater mechanical strength and withstands higher loads than the single-screw systems [18–20]. Yet, the performances between the dual-screw InterTAN and the single-screw PFNA/PFNA-II or Gamma3 remain inconclusive. Here, we performed a systematic review and meta-analysis comparing dual-screw versus single-screw cephalomedullary nails in aspects of perioperative parameters, clinical and radiological outcomes, complications, and mortality, aiming to provide evidence for the surgical choice of IFF.

## Methods

### Literature search

This systematic review and meta-analysis were performed following the Preferred Reporting Items for Systematic reviews and Meta-Analyses (PRISMA) guideline (Additional files 1 and 2). Electronic literature databases, including PubMed, EMBASE, Web of Science, and Cochrane Library, were searched for eligible studies comparing the effectiveness and safety of dual-screw cephalomedullary nail (InterTAN) and single-screw cephalomedullary nail (PFNA, PFNA-II, Gamma3) in IFFs from the inception to April 30, 2023. The search terms were used: (“INTERTAN” OR “intertrochanteric antegrade nail”) AND (“hip fracture” OR “Intertrochanteric femoral fractures” OR “intertrochanteric fracture”). There was no language restriction. References of identified articles were further reviewed for eligibility of meta-analysis.

### Selection of eligible studies

Eligible studies were selected by two independent researchers according to the PICOS framework. Population (P): patients with IFF. Intervention (I): InterTAN nails. Comparison (C): PFNA, PFNA-II, or Gamma3 nail. Outcome (O): perioperative parameters, clinical and radiological outcomes, complications, mortality. Study design (S): randomized controlled trial (RCT), prospective or retrospective observational study. There was no language restriction. Review articles, meta-analyses, biomechanical research, experimental studies, and duplicates were excluded.

### Outcomes

Perioperative parameters included operative time (minutes), intraoperative blood loss (mL), fluoroscopy time (minutes), and length of hospital stay (days). Clinical and radiological outcomes included Harris Hip Score (HHS) at 6 months, 1 year after surgery, and at the last follow-up, time to union/healing (weeks), femoral neck shortening (mm), and time to full bearing (weeks). Complications included implant failures, varus collapse, femoral shaft fracture, screw migration, non-union, cut-out, hip and thigh pain, deep venous thrombosis, infection, and revision/reoperation. Mortality after the operation was also analyzed.

### Data extraction

Two independent researchers extracted the following information from included studies: first author, publication year, study design, comparator, sample size, fracture type (AO/OTA 31A1, A2, and A3), mean age, percentage of males, duration of follow-up, perioperative parameters, clinical and radiological outcomes, and complications.

### Methodology assessment

The risk of bias of RCTs was evaluated according to Cochrane Collaboration’s tool for assessing risk of bias [21], which graded selection, performance, detection, attrition, reporting, and other bias at low, high, or unclear risk. The quality of observational studies was assessed using Good Research for Comparative Effectiveness (GRACE) checklist containing 11 items in terms of the use of concurrent comparators, equivalent measurement of outcomes in different groups, collection of data on confounders and effect modifiers, risk of immortal time bias, and reporting of sensitivity analysis [22]. Any disagreement regarding literature selection, data extraction, risk of bias, and quality assessment was resolved by a third researcher.

### Statistical analysis

The between-study heterogeneity was evaluated using I^2^ statistics and the Q test. The model for quantitative analysis was determined according to heterogeneity. *I*^2^ > 50% with *Q* test *P* value < 0.10 indicated substantial heterogeneity, and the random-effect model was applied. Otherwise, the fixed-effect model was used. Mean difference (MD) and corresponding 95% confidence interval (95%CI) were calculated for continuous variables, while risk ratio (RR) with 95%CI was estimated for dichotomous variables. For quantitative analysis comprising 10 or more eligible studies, further subgroup analysis was performed in terms of the comparator (PFNA/PFNA-II, Gamma3), study design (RCT, observational study), and fracture type (unstable type only, mixed types). Sensitivity analysis was conducted using the leave-one-out method to assess the robustness of pooled results. Publication bias was indicated by the symmetry of the funnel plot and assessed by Egger’s test. *P* < 0.05 was considered to be statistically significant.

## Results

### Characteristics of studies included in the meta-analysis

**Fig. 1:**
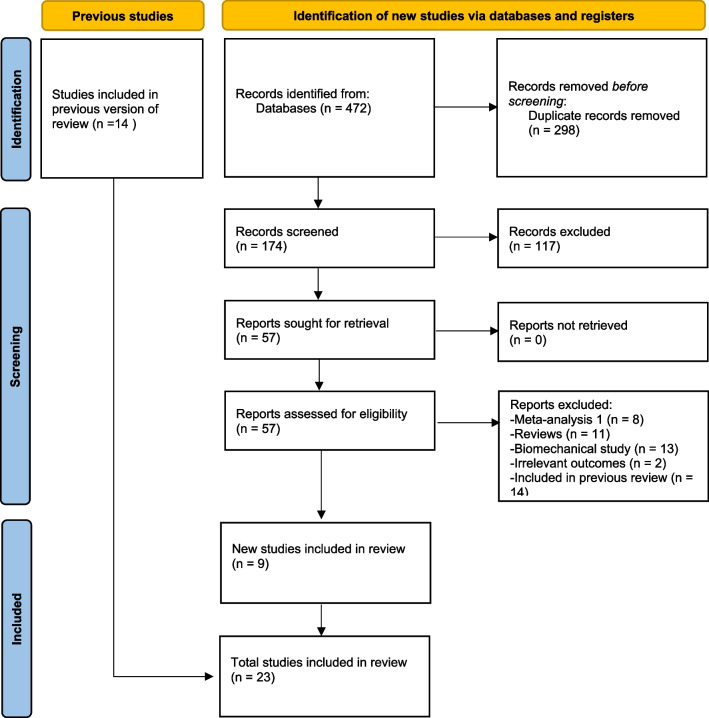
Flowchart of literature search

Literature search yielded 174 unique articles after removing duplicates, and 117 articles not related to the topic were excluded. Among the remaining 57 articles that were reviewed for full texts, 34 were excluded as they were meta-analyses, review articles, biomechanical studies or provided irrelevant outcomes (Additional file 3: Table S1). Finally, 23 studies comprising 3566 patients were included for meta-analysis [23–45] (Fig. 1). Among them, 1832 patients were treated with InterTAN, 528 patients from 6 studies were treated with Gamma3 as the comparator, and 1206 patients from 17 studies were treated with PFNA/PNFA-II as the comparator. There were 310 stable (AO/OTA 31A1) and 1394 unstable (AO/OTA 31A2/A3) fractures in the InterTAN group and 192 stable and 1395 unstable fractures in the comparator group. Fourteen studies enrolled unstable fractures only, 7 studies included both stable and unstable types [23, 24, 28–30, 38, 45], and 2 studies did not mention fracture types [32, 34]. As to study design, 5 were RCTs [23, 26, 30, 41, 44], 2 were prospective observational studies [24, 33] and the others were retrospective studies. The included studies had diverse durations of follow-up, among which 3 had less than 1 year of mean follow-up duration [26, 28, 32]. Two studies were published in Chinese [32, 40] and the others were in English. The characteristics of all studies are summarized in Table 1. The outcomes reported in each study are listed in Additional file 3: Table S2.

**Table 1:** Characteristics of all studies included in the meta-analysis

Study	Design	Comparator	Sample size^#^	AO/OTA 31 (A1/A2/A3)		Age (years)^#^	Male (%)^#^	Follow-up (months)
				InterTAN	Comparator			
Zhang [41]	RCT	PFNA-II	57/56	0/45/12	0/45/11	72.9 ± 7.6/72.4 ± 8.7	40.4/33.9	18.36 ± 5.83 (range 12–30)
Wang [32]	RS	PFNA	20/36	NR	NR	73.5 ± 11.3/76.8 ± 9.5	55/47.2	4.1 (range 2.5–14)
Wu [33]	PS	Gamma3	87/174	0/72/15	0/146/28	71.4 ± 9.7/72.6 ± 8.6	77/75.3	12
Seyhan [30]	RCT	PFNA	32/43	7/13/12	11/16/16	75.34 ± 13.52/75.91 ± 13.77	75/74.4	19.4 (range 12–60)
Yu [35]	RS	PFNA-II	75/72	0/40/35	0/35/37	75.2 ± 8.8/74.2 ± 9.1	46.7/44.4	20 (range 16–26)
Zehir [36]	RS	PFNA	102/96	0/93/9	0/92/4	76.86 ± 6.74/77.22 ± 6.82	38.2/38.5	Median 16 (range 1–46)
Berger-Groch [23]	RCT	Gamma3	55/49	14 stable, 31 unstable	14 stable, 31 unstable	81.6 ± 9.4/82.0 ± 9.2	21.8/24.5	60
Hopp [26]	RCT	Gamma3	39/39	0/28/11	0/26/13	82.70 ± 7.06/80.73 ± 8.44	17.9/33.3	5.95 ± 3.91
Su [44]	RCT	Gamma3	50/50	0/40/10	0/41/9	70.1 ± 9.2/71.3 ± 8.7	42/38	12
Zhang [38]	RS	PFNA-II	86/88	37/49/0	42/46/0	72.7 ± 7.6/7.6 ± 6.3	34.9/38.6	40 (range 38–60)
Serrano [29]	RS	Gamma3	283/130	155 stable, 128 unstable	79 stable, 51 unstable	76^&^	33^&^	12
Zhang [39]	RS	PFNA	144/139	0/144/0	0/139/0	76.1^&^	44.4/38.1	38.8 (range 36–43)
Zhang [40]	RS	PFNA	49/64	0/37/12	0/48/16	74.2 ± 5.4/73.3 ± 6.5	40.8/39.1	16.3 ± 1.2 (range 14–18)
Gavaskar [25]	RS	PFNA-II	50/50	0/31/19	0/31/19	77 ± 7/78 ± 8	42/42	12
Zhang [37]	RS	PFNA	162/165	0/162/0	0/164/0	72.3 ± 4.6^&^	45.1/47	43.5 (range 38–48)
Imerci [27]	RS	PFNA	36/33	0/0/36	0/0/33	57.86 ± 22.00/54.64 ± 18.94	63.9/57.6	Minimum 12
Duramaz [24]	PS	PFNA-II	86/100	34/32/20	28/49/23	61.5 ± 15.8/60.01 ± 16.6	43.6^&^	25.9 ± 2.5
Ulku [45]	RS	PFNA	12/16	1/4/7	2/5/9	65^&^	28.6^&^	19.4 (range 12–60)
Zhao [42]	RS	Gamma3	79/86	0/79/0	0/86/0	75.56 ± 14.89/73.61 ± 16.22	31.6/31.4	12
Su [31]	RS	PFNA	41/34	0/37/4	0/32/2	68.61 ± 6.7/66.97 ± 4.79	31.7/32.4	Minimum 12
Polat [28]	RS	PFNA	144/65	62 stable, 82 unstable	16 stable, 49 unstable	80 ± 9.7/85.2 ± 4.5	29.2/26.2	3
Zhu [43]	RS	PFNA-II	45/43	0/27/18	0/25/18	69.13 ± 4.88/68.30 ± 5.35	57.8/60.5	NR
Yalin [34]	RS	PFNA	98/107	NR	NR	76.6 ± 9.64/77.81 ± 6.64	48/45.8	Minimum 12

^#^ InterTAN/Comparator

^&^Total population

*NR*: not reported; *PFNA*: proximal femoral nail anti-rotation; *PS*: prospective study; *RCT*: randomized controlled trial; *RS*: retrospective study

### Methodology assessment

Among the 5 RCTs, 1 used computer-generated sequences for randomization [41] and was judged to be at low risk of bias for this domain. The other 4 trials did not specify the method of random sequence generation and were deemed at unclear risk of bias. Two studies used numbered and blinded envelopes and were deemed at low risk of allocation concealment [23, 26]. The other 3 studies used sealed envelopes and were judged to be at unclear risk of bias for this domain. Since the surgeons could not be blinded, all trials were judged to be at unclear risk of bias relating to blinding of participants and personnel. Blinding of outcome assessment was stated in 1 trial, which was deemed at low risk of bias [23]. The authors of all RCTs reported no conflict of interest and no financial relationship with devices companies. The results of risk of bias assessment are summarized in Additional file 3: Table S3. The average GRACE score of the other 18 non-randomized trials was 9.4 (range 7–10), suggesting good methodological quality of these studies (Additional file 3: Table S4).

### Perioperative parameters

Perioperative parameters, including operative time, fluoroscopy time, intraoperative blood loss, and length of hospital stay, were reported in 18 (2602 fractures), 10 (1401 fractures), 14 (2190 fractures), and 9 studies (1314 fractures), respectively. Meta-analysis using a random-effect model demonstrated, compared to single-screw cephalomedullary nail (PFNA/PFNA-II, Gamma3), InterTAN had significantly longer operative time (MD = 5.93 min, 95%CI 0.19 to 11.67, *P* = 0.043, Additional file 4: Figure S1), longer fluoroscopy time (MD = 0.68 min, 95%CI 0.32 to 1.04, *P* < 0.001, Additional file 4: Figure S2) and more intraoperative blood loss (MD = 18.19 mL, 95%CI 8.03 to 28.34, *P* < 0.001, Additional file 4: Figure S3). Yet, the length of hospital stay did not differ between both groups (MD = − 0.43 days, 95%CI  − 1.12 to 0.27, *P* = 0.232, Additional file 4: Figure S4).

### Clinical and radiological outcomes

**Fig. 2:**
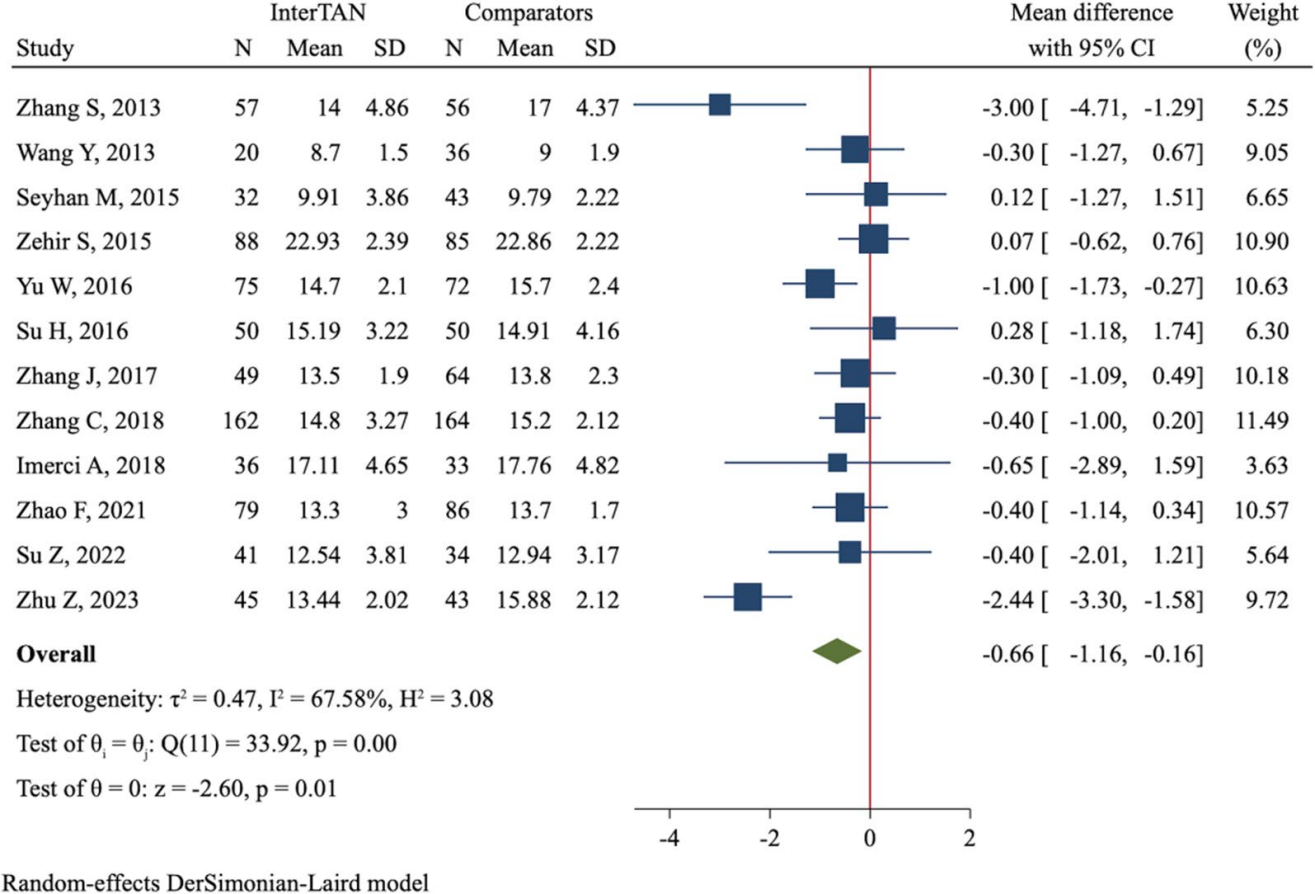
Forest plot of meta-analysis of time to union/healing

Twelve studies involving 1500 fractures compared the time to union/healing between dual-screw and single-screw cephalomedullary nails. Meta-analysis applying a random-effect model showed patients treated with InterTAN achieved union/healing significantly earlier than those treated with PFNA/PFNA-II or Gamma3 nails (MD = − 0.66 days, 95%CI  − 1.16 to  − 0.16, *P* = 0.009, Fig. 2). There were no significant differences between both groups in terms of femoral neck shortening (MD = − 0.76 mm, 95%CI  − 1.69 to 0.17, *P* = 0.110, Additional file 4: Figure S5) and time to full bearing (MD = 0.18 weeks, 95%CI  − 0.20 to 0.56, *P* = 0.353, Additional file 4: Figure S6).

**Fig. 3:**
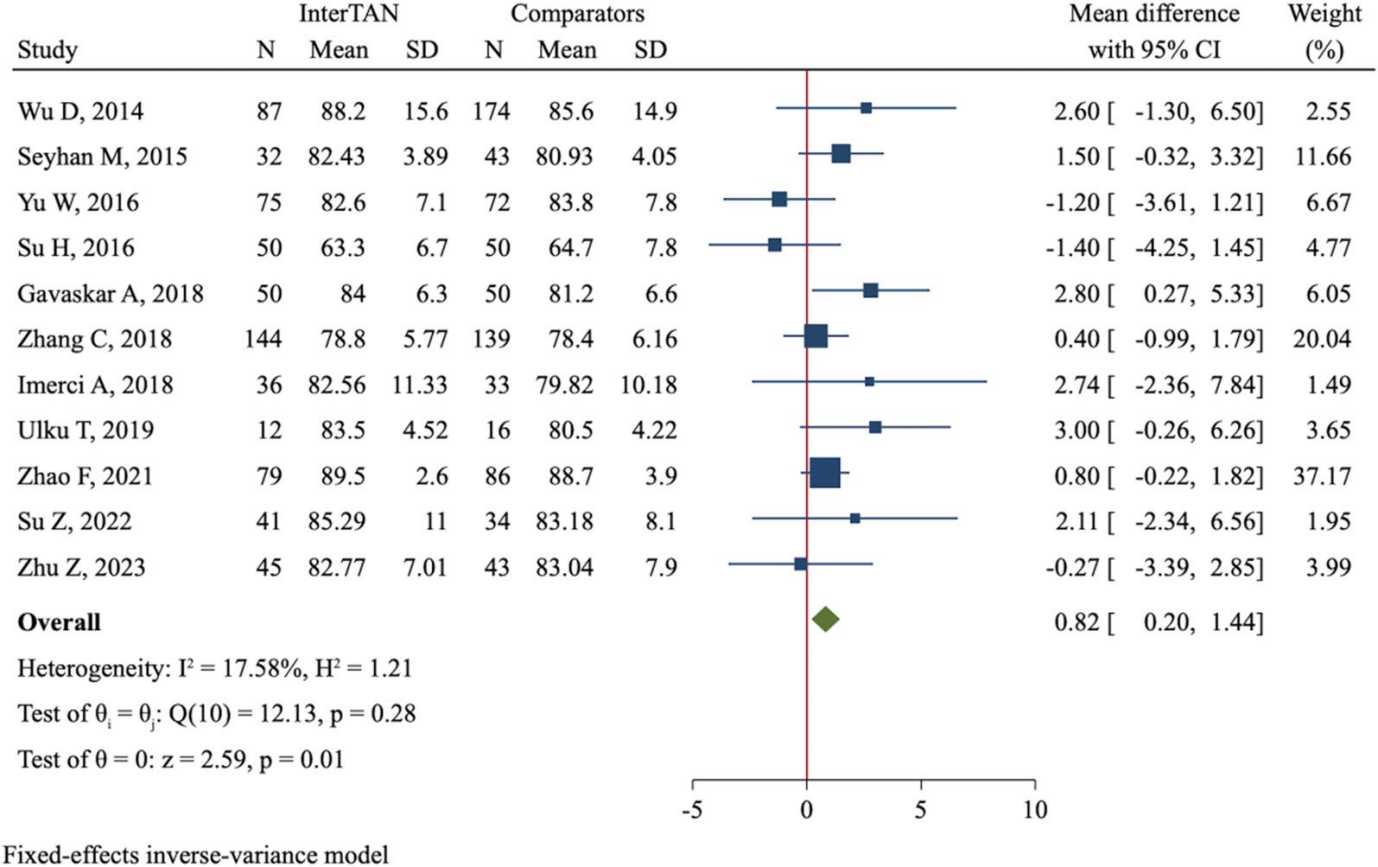
Forest plot of meta-analysis of 1-year Harris Hip Score

HHS was assessed at three different time points after surgery: 6 months, 1 year, and the last follow-up. Only 4 studies (611 patients) reported HHS at 6 months after surgery, which did not show a significant difference through meta-analysis (MD = 1.49, 95%CI  − 0.59 to 3.58, *P* = 0.161, Additional file 4: Figure S7). The 1-year HHS was compared in 11 studies (1391 patients). The between-study heterogeneity was low (*I*^2^ = 17.6%, *P* = 0.276), and thus a fixed-effect model was applied. The InterTAN group had a significantly higher 1-year HHS compared with PFNA/PFNA-II and Gamma3 nails (MD = 0.82, 95%CI 0.20–1.44, *P* = 0.010, Fig. 3). However, the difference in HHS was not significant at the last follow-up (MD = 0.62, 95%CI  − 0.73 to 1.97, *P* = 0.365, Additional file 4: Figure S8).

### Complications

**Fig. 4:**
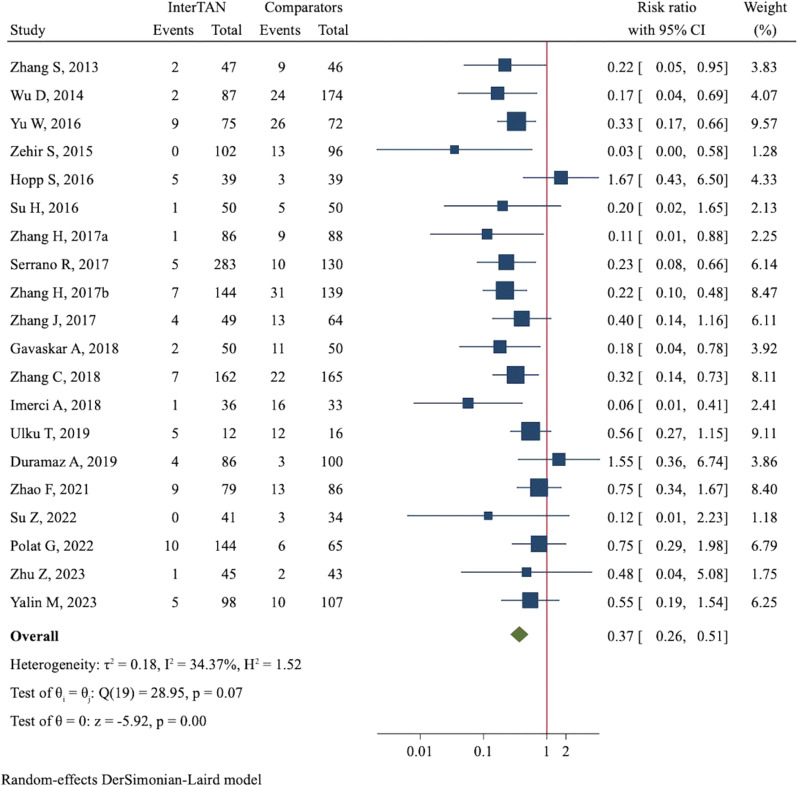
Forest plot of meta-analysis of implant failures

Implant failures, which included femoral shaft fracture, cut-out, screw migration, varus collapse, implant breakage, and z-effect, were documented in 20 studies. The incidence of implant failures was only 4.7% (80/1715) in the InterTAN group compared with 15.1% (241/1597) in the control group. Thus, InterTAN significantly reduced the risk of implant failures (RR = 0.37, 95%CI 0.26 to 0.51, *P* < 0.001, Fig. 4). As to the specific failures, InterTAN was shown to significantly reduce the risk of femoral shaft fracture (RR = 0.22, 95%CI 0.12 to 0.42, *P* < 0.001, Additional file 4: Figure S9), cut-out (RR = 0.30, 95%CI 0.19 to 0.47, P < 0.001, Additional file 4: Figure S10), screw migration (RR = 0.21, 95%CI 0.10 to 0.43, *P* < 0.001, Additional file 4: Figure S11) and varus collapse (RR = 0.33, 95%CI 0.18 to 0.62, *P* < 0.001, Additional file 4: Figure S12) through meta-analyses in a fixed-effect model.

**Fig. 5:**
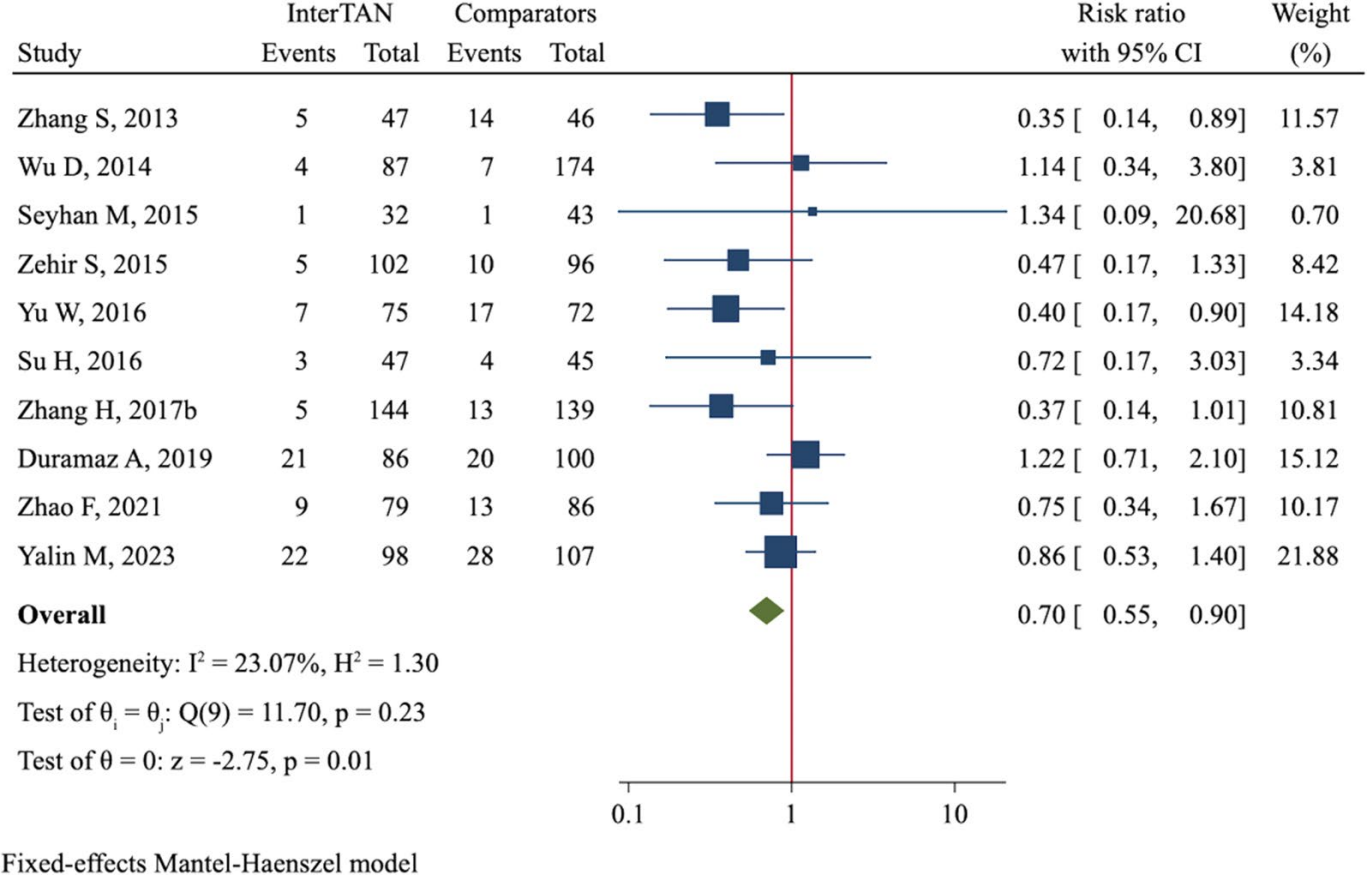
Forest plot of meta-analysis of hip and thigh pain

**Fig. 6:**
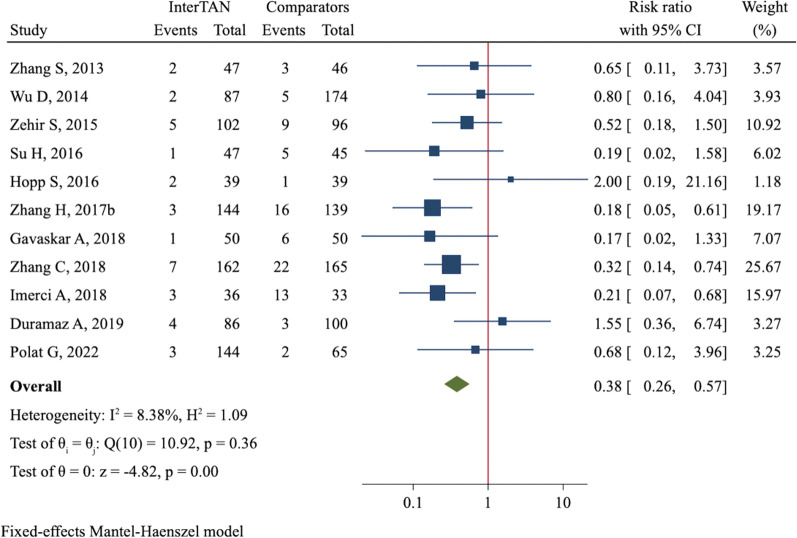
Forest plot of meta-analysis of revision/reoperation

Hip and thigh pain was reported in 10 studies comprising 1705 fractures. The overall incidence in the InterTAN group was 10.3% (82/797), which was significantly lower than 14.0% (127/908) in the control group, demonstrating a reduced risk of hip and thigh pain in patients treated with InterTAN (RR = 0.70, 95%CI 0.55 to 0.90, *P* = 0.006, Fig. 5). Due to various complications, 33 (3.5%) out of 944 patients in the InterTAN group and 85 (8.9%) out of 952 patients in the control group received revision or reoperation. Meta-analysis using a fixed-effect model showed InterTAN might significantly reduce the risk of revision/reoperation as compared with PFNA/PFNA-II or Gamma3 (RR = 0.38, 95%CI 0.26 to 0.57, *P* < 0.001, Fig. 6). Yet, through meta-analyses, we did not observe a significant difference in incidences of non-union (RR = 0.79, 95%CI 0.31 to 2.03, *P* = 0.619, Additional file 4: Figure S13), infection (RR = 1.31, 95%CI 0.77 to 2.25, *P* = 0.318, Additional file 4: Figure S14) and deep venous thrombosis (RR = 0.99, 95%CI 0.63 to 1.55, *P* = 0.955, Additional file 4: Figure S15).

### Mortality

Six studies (1063 patients) reported mortality after surgery, of which 1 reported in-hospital mortality [36], 2 reported 1-year mortality [34, 42] and 3 reported mortality at last follow-up [23, 39, 40]. Meta-analysis using a fixed-effect model suggested no significant difference in mortality between InterTAN and the comparators (RR = 0.88, 95%CI 0.71 to 1.10, *P* = 0.266, Additional file 4: Figure S16).

### Subgroup and sensitivity analyses

Subgroup analyses were performed in outcomes involving 10 or more available studies, which included operative time, intraoperative blood loss, fluoroscopy time, 1-year HHS, time to union/healing, implant failures, cut-out, hip and thigh pain, and revision/reoperation (Additional file 3: Table S5). Compared with PFNA/PFNA-II, InterTAN had significantly higher 1-year HHS, shortened time to union/healing, and reduced risks of implant failures, cut-out, hip and thigh pain, and revision/reoperation. Among studies only containing unstable fractures (AO/OTA 31A2/A3), InterTAN could significantly reduce the risk of various complications. As to study design, the significant differences in perioperative parameters, 1-year HHS, and risk of complications were mainly observed in the subgroup of observational studies but not in the RCT subgroup.

Sensitivity analysis using the “Leave-one-out” method showed the differences in femoral neck shortening (MD = − 1.39 mm, 95%CI − 1.99 to − 0.78, *P* < 0.001) and 6-month HHS (MD = 2.17, 95%CI 0.18 to 4.16, *P* = 0.033) became significant after omitting Zhang et al. study [41] and Zhu et al. study [43], respectively. Furthermore, sensitivity analysis demonstrated the pooled result of operative time was not robust as the difference became insignificant after the individual omission of several studies (Additional file 4: Figure S17). In addition, if we excluded two outliners [35, 37], the between-study heterogeneity of time to union/healing reduced from 67.6% to 0. Meta-analysis using a fixed-effect model revealed a significantly shortened time to union/healing in the InterTAN group (MD = -0.35 weeks, 95%CI − 0.63 to − 0.07, *P* = 0.014). For the other outcomes, no single study had a significant impact on the pooled results.

### Publication bias

**Fig. 7:**
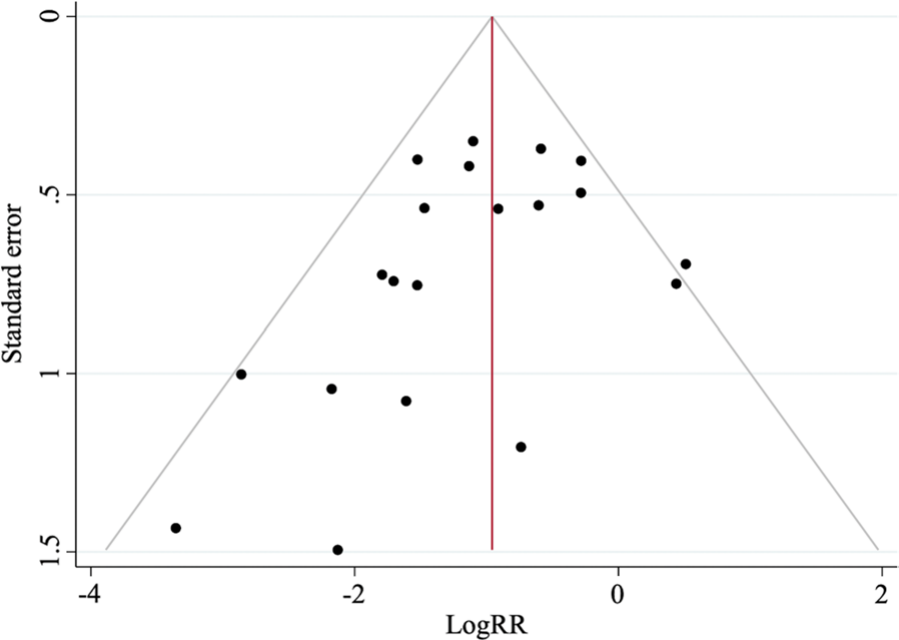
Funnel plot of meta-analysis of implant failure

We observed asymmetry of funnel plots, and Egger’s test suggested potential publication bias of fluoroscopy time (*P* < 0.001), femoral neck shortening (*P* = 0.008), implant failures (*P* = 0.058, Fig. 7) and cut-out (*P* = 0.052). There was no evidence of publication bias in the other outcomes.

## Discussion

Internal fixation using cephalomedullary nails is more and more widely used in the treatment of IFF, but which one of the commonly used screws, InterTAN, PFNA/PFNA-II, or Gamma3, has clinical advantages over the other nails is still in controversy. The present study, having the largest sample size, comprehensively compares the integrated dual-screw InterTAN with single-screw cephalomedullary nail (PFNA/PFNA-II, Gamma3) for the treatment of IFF in aspects of perioperative parameters, clinical and radiological outcomes, complications and mortality. We find, although conferring longer operative and fluoroscopy times and more intraoperative blood loss, InerTAN achieves earlier union/healing and improved functional scores, and reduces the risk of various complications including implant failures, femoral shaft fracture, cut-out, screw migration, varus collapse, hip and thigh pain, and revision/reoperation. Besides, both dual-screw and single-screw implants have comparable performance in the length of hospital stay, complications such as non-union, infection and deep venous thrombosis, and mortality. Our meta-analysis suggests InterTAN may be a better surgical choice than single-screw cephalomedullary nails for the treatment of IFF.

InterTAN inserts two screws into the head-neck fragment and is considered to be a more invasive surgery than the single-screw systems. Thus, a longer operative time is usually needed in InterTAN, which subsequently results in more fluoroscopy time and intraoperative blood loss. This is consistent with the results of our meta-analysis of perioperative parameters. Yet, two studies reported a shorter operative time in the InterTAN group [34, 44], and several trials found comparable operative time between both groups. The diversity of these perioperative parameters among different studies may be owing to the difference in skill levels of surgeons and fracture complexity.

Several biomechanical comparisons have been performed between InterTAN, PFNA/PFNA-II, and Gamma3. Luo et al. found comparable biomechanical stability of InterTAN and Gamma3 in the stable fracture model but significantly higher failure load and torque of the InterTAN group in the unstable fracture model [46]. Another study using a cadaveric hemipelvis biomechanical model revealed greater stability and resistance to femoral head rotation and varus collapse of the InterTAN construct [20]. Huang et al. observed significantly increased strength, stiffness, and resistance torque when comparing InterTAN to PFNA [18]. Therefore, the integrated dual-screw InterTAN provides greater and more stable intersegmental compression and firmer fixation, which allows for the maintenance of reduction and the stability required for fractures to heal, than the single-screw nail. These advantages may explain our findings of significantly reduced risk of complications, such as cut-out and varus collapse, the decreased need for revision/reoperation, and a shorter time to union/healing in the InterTAN group.

Compared with previously performed meta-analyses [47–52], our study has several strengths and adds new findings. Firstly, the present meta-analysis has the largest sample size with over 3500 patients from 23 included trials, which is 1.35-fold of the sample size of a recent similar meta-analysis [50]. Secondly, our study is a more comprehensive comparison of dual-screw versus single-screw cephalomedullary nails in all IFFs by the evaluation of multiple parameters and outcomes. More outcomes, such as time to full bearing, infection, deep venous thrombosis, and mortality, were analyzed in present meta-analysis. Thirdly, we have performed detailed subgroup analyses by comparator, study design, and fracture type. We find the priority of InterTAN to PFNA/PFNA-II in perioperative parameters, clinical outcomes, and complications. Yet, the priority to Gamma3 is lacking in most of the outcomes except cut-out, which may be due to the small sample size. In unstable fractures (AO/OTA 31A2 and A3), InterTAN has prior performance than single-screw nails. Finally, our analysis, for the first time, shows a significantly shorter time to union/healing, even after excluding two outliners, and a higher 1-year HHS in the InterTAN group than in the single-screw nails as more available studies are included. Different from the most recent meta-analysis including only 15 studies [50], our meta-analysis finds significantly reduced risks of implant failures, femoral shaft fracture, and varus collapse in the InterTAN group. Moreover, we have compared the mortality after surgery between dual-screw and single-screw nails by including 6 available studies for the first time. Despite less complications and better clinical and functional performances, the mortality of InterTAN does not differ from that of single-screw nails.

The major limitation of our meta-analysis is that the majority of included trials are retrospective observational studies that only have a low level of evidence. Only 5 RCTs with a high level of evidence are available and included. As indicated by the subgroup analysis, the significant differences in outcomes are mainly found in the subgroup of observational studies but not in the subgroup of randomized trials. Thus, the certainty of evidence is moderate or below. More well-designed RCTs are warranted to further confirm these findings in the future.

## Conclusions

In conclusion, our meta-analysis demonstrates InterTAN has better performances than single-screw cephalomedullary nails (PFNA/PFNA-II, Gamma3) in terms of earlier union/healing, higher 1-year HHS and reduced risk of various complications such as implant failures, hip and thigh pain, and revision/reoperation. InterTAN should be a preferable choice for the treatment of IFF, especially unstable fractures.

### Supplementary Information

**Additional file 1**. PRISMA 2020 Checklist.

**Additional file 2**. PRISMA 2020 for Abstract Checklist.

**Additional file 3**. **Table S1** List of excluded articles and their reasons. **Table S2** Outcomes reported in studies of the meta-analysis. **Table S3** Risk of bias assessment of randomized controlled trials. **Table S4** Quality assessment of observational studies using GRACE checklist. **Table S5** Results of subgroup analyses.

**Additional file 4**. **Figure S1** Forest plot of meta-analysis of operative time. **Figure S2** Forest plot of meta-analysis of fluoroscopy time. **Figure S3** Forest plot of meta-analysis of intraoperative blood loss. **Figure S4** Forest plot of meta-analysis of length of hospital stay. **Figure S5** Forest plot of meta-analysis of femoral neck shortening. **Figure S6** Forest plot of meta-analysis of time to full bearing. **Figure S7** Forest plot of meta-analysis of 6-month Harris Hip Score. **Figure S8** Forest plot of meta-analysis of Harris Hip Score at last follow-up. **Figure S9** Forest plot of meta-analysis of femoral shaft fracture. **Figure S10** Forest plot of meta-analysis of cut-out. **Figure S11** Forest plot of meta-analysis of screw migration. **Figure S12** Forest plot of meta-analysis of varus collapse. **Figure S13** Forest plot of meta-analysis of non-union. **Figure S14** Forest plot of meta-analysis of infection. **Figure S15** Forest plot of meta-analysis of deep venous thrombosis. **Figure S16** Forest plot of meta-analysis of mortality. **Figure S17** Sensitivity analysis of operative time using the “Leave-one-out” method.

## Data Availability

All data generated or analyzed during this study are included in this published article and its supplementary information files.

## Abbreviations

IFF: Intertrochanteric femoral fracture
InterTAN: Intertrochanteric antegrade nail
PFNA: Proximal femoral nail anti-rotation
HHS: Harris Hip Score
MD: Mean difference
RR: Risk ratio
