# Supplementary material for: Dual-screw versus single-screw cephalomedullary nails for intertrochanteric femoral fractures: a systematic review and meta-analysis
Source: J Orthop Surg Res. 2023 Aug 20;18:607. doi: 10.1186/s13018-023-04103-x (PMC10440877; doi:10.1186/s13018-023-04103-x)
Supplement: Supplementary file 3 — Additional file 3. Table S1 List of excluded articles and their reasons. Table S2 Outcomes reported in studies of the meta-analysis. Table S3 Risk of bias assessment of randomized controlled trials. Table S4 Quality assessment of observational studies using GRACE checklist. Table S5 Results of subgroup analyses. [file 13018_2023_4103_MOESM3_ESM.docx]

Table S1. List of excluded articles and their reasons.

| Author, year | Title | Reasons for exclusion | PMID |
| --- | --- | --- | --- |
| Hoffmann S, 2013 | Biomechanical evaluation of interlocking lag screw design in intramedullary nailing of unstable pertrochanteric fractures | Biomechanical study | 23860133 |
| Huang Y, 2013 | A comparative biomechanical study of proximal femoral nail (InterTAN) and proximal femoral nail antirotation for intertrochanteric fractures | Biomechanical study | 24091417 |
| Nuchtern J, 2014 | Malpositioning of the lag screws by 1- or 2-screw nailing systems for pertrochanteric femoral fractures: a biomechanical comparison of gamma 3 and intertan | Biomechanical study | 24751606 |
| Chen Y, 2014 | Comparative biomechanical study of reversed less invasive stabilization system and proximal femoral nail antirotation for unstable intertrochanteric fractures | Biomechanical study | 25430461 |
| Konstantinidis L, 2015 | Sliding of the load carrier in third-generation intramedullary nails for proximal femur fractures: an in vitro mechanical comparison study | Biomechanical study | 25617021 |
| Knobe M, 2015 | Locked minimally invasive plating versus fourth generation nailing in the treatment of AO/OTA 31A2.2 fractures: A biomechanical comparison of PCCP(®) and Intertan nail(®) | Biomechanical study | 25997559 |
| Nyholm A, 2016 | Lacking evidence for performance of implants used for proximal femoral fractures - A systematic review | Review | 26803696 |
| Santoni B, 2016 | Comparison of Femoral Head Rotation and Varus Collapse Between a Single Lag Screw and Integrated Dual Screw Intertrochanteric Hip Fracture Fixation Device Using a Cadaveric Hemi-Pelvis Biomechanical Model | Biomechanical study | 27003028 |
| Sambandam S, 2016 | Intertrochanteric fractures: a review of fixation methods. | Review | 27028746 |
| Baldwin P, 2016 | Controversies in Intramedullary Fixation for Intertrochanteric Hip Fractures | Review | 27437614 |
| Socci A, 2017 | Implant options for the treatment of intertrochanteric fractures of the hip: rationale, evidence, and recommendations | Review | 28053268 |
| Yang X, 2017 | Investigation of perioperative hidden blood loss of unstable intertrochanteric fracture in the elderly treated with different intramedullary fixations | Irrelevant outcomes (perioperative hidde blood loss) | 28693817 |
| Rosa N, 2017 | Recent developments on intramedullary nailing: a biomechanical perspective | Review | 29090836 |
| Ma J, 2017 | Comparison of clinical outcomes with InterTan vs Gamma nail or PFNA in the treatment of intertrochanteric fractures: A meta-analysis | Meta-analysis | 29162931 |
| Nherera L, 2018 | Comparison of a twin interlocking derotation and compression screw cephalomedullary nail (InterTAN) with a single screw derotation cephalomedullary nail (proximal femoral nail antirotation): a systematic review and meta-analysis for intertrochanteric fractures | Meta-analysis | 29499715 |
| Nherera L, 2018 | Comparing the costs and outcomes of an integrated twin compression screw (ITCS) nail with standard of care using a single lag screw or a single helical blade cephalomedullary nail in patients with intertrochanteric hip fractures | Irrelevant outcomes (cost) | 30165881 |
| Shu W, 2018 | Comparison of effects of four treatment methods for unstable intertrochanteric fractures: A network meta-analysis | Meta-analysis | 30445196 |
| Pradhananga M, 2018 | Comparison of intramedullary nails in the treatment of intertrochanteric fracture - a review | Review | Link^#^ |
| Santoni B, 2019 | Biomechanical Investigation of an Integrated 2-Screw Cephalomedullary Nail Versus a Sliding Hip Screw in Unstable Intertrochanteric Fractures | Biomechanical study | 30562248 |
| Li J, 2019 | Evolving concept in treatment of intertrochanteric fractures and development of internal fixation devices | Review | 30644252 |
| Cha Y, 2019 | Biomechanical Evaluation of Internal Fixation of Pauwels Type III Femoral Neck Fractures: A Systematic Review of Various Fixation Methods | Review | 30838102 |
| Yu C, 2019 | PFNA and InterTAN intramedullary nailing in elderly patients with femoral intertrochanteric fractures: a Meta analysis | Meta-analysis | 30884927 |
| Hao L, 2019 | Finite element analysis of InterTan for the treatment of femoral intertrochanteric fractures | Biomechanical study | 30884938 |
| Cipollaro L, 2019 | Single- versus double-integrated screws in intramedullary nailing systems for surgical management of extracapsular hip fractures in the elderly: a systematic review | Review | 31172929 |
| Liu W, 2020 | Comparison of clinical outcomes with proximal femoral nail anti-rotation versus InterTAN nail for intertrochanteric femoral fractures: a meta-analysis | Meta-analysis | 33121518 |
| Luo W, 2020 | Biomechanical Comparison of INTERTAN Nail and Gamma3 Nail for Intertrochanteric Fractures | Biomechanical study | 33215879 |
| Date A, 2020 | Comparison of clinical and radiological outcomes in intertrochanteric fractures treated with InterTAN nail against conventional cephalomedullary nails: a systematic review | Review | 33437510 |
| Onggo JR, 2022 | Integrated dual lag screws versus single lag screw cephalomedullary nail constructs: a meta-analysis and systematic review | Meta-analysis | 33566701 |
| Pastor T, 2022 | Biomechanical analysis of recently released cephalomedullary nails for trochanteric femoral fracture fixation in a human cadaveric model | Biomechanical study | 34748055 |
| Quartley M, 2022 | Stabilisation of AO OTA 31-A unstable proximal femoral fractures: Does the choice of intramedullary nail affect the incidence of post-operative complications? A systematic literature review and meta-analysis | Meta-analysis | 35151468 |
| Zhang W, 2022 | Biomechanical comparison of the femoral neck system versus InterTan nail and three cannulated screws for unstable Pauwels type III femoral neck fracture | Biomechanical study | 35689282 |
| Wang Y, 2022 | Finite Element Analysis of Proximal Femur Bionic Nail (PFBN) Compared with Proximal Femoral Nail Antirotation and InterTan in Treatment of Intertrochanteric Fractures | Biomechanical study | 35848160 |
| Petfield J, 2022 | Tips and tricks to avoid implant failure in proximal femur fractures treated with cephalomedullary nails: a review of the literature | Review | 35949270 |
| Wang W, 2023 | Meta-analysis of InterTan, PFNA and PFNA-II internal fixation for the treatment of unstable intertrochanteric fractures in elderly individuals | Meta-analysis | 37294985 |

^#^http://ijsit.com/admin/ijsit_files/COMPARISON%20OF%20INTRAMEDULLARY%20NAILS%20IN%20THE%20TREATMENT%20OF%20INTERTROCHANTERIC%20FRACTURE%20-%20A%20REVIEW_IJSIT_7.2.17.pdf.

Table S2. Outcomes reported in studies of the meta-analysis.

| Study | Perioperative parameters | | | |  | Clinical and radiological outcomes | | | | | |  | Complications | | | | | | | | | | Mortality |
| --- | --- | --- | --- | --- | --- | --- | --- | --- | --- | --- | --- | --- | --- | --- | --- | --- | --- | --- | --- | --- | --- | --- | --- |
|  | 1 | 2 | 3 | 4 |  | 5 | 6 | 7 | 8 | 9 | 10 |  | 11 | 12 | 13 | 14 | 15 | 16 | 17 | 18 | 19 | 20 |  |
| Zhang S, 2013 | + | + | + | + |  |  |  | + | + | + |  |  | + |  | + | + |  | + | + | + | + | + |  |
| Wang Y, 2013 | + | + |  |  |  |  |  | + | + |  |  |  | NE |  |  |  |  |  |  |  |  |  |  |
| Wu D, 2014 | + | + | + | + |  |  | + |  |  | + |  |  | + |  | + |  | + | + | + | + | NE | + |  |
| Seyhan M, 2015 | + |  | + |  |  |  | + |  | + |  | + |  | NE | NE |  |  | NE | NE | + |  | NE | NE |  |
| Yu W, 2016 | + | + | + | + |  |  | + |  | + | + |  |  | + | + | + | + |  | + | + |  |  |  |  |
| Zehir S, 2015 | + | + | + |  |  |  |  | + | + |  |  |  | + |  |  | + |  | + | + | + | + | + | + |
| Berger-Groch J, 2016 | + |  |  | + |  |  |  |  |  |  |  |  |  |  |  |  |  |  |  |  |  |  | + |
| Hopp S, 2016 | + | + |  | + |  |  |  | + |  |  |  |  | + |  |  |  |  |  |  |  |  | + |  |
| Su H, 2016 | + | + |  |  |  |  | + |  | + | + |  |  | + |  |  |  | NE | + | + | + | NE | + |  |
| Zhang H, 2017a |  |  |  |  |  |  |  |  |  |  |  |  | + |  | + | + | + | + |  |  |  |  |  |
| Serrano R, 2017 |  |  |  |  |  |  |  |  |  |  |  |  | + |  |  |  |  | + |  |  |  |  |  |
| Zhang H, 2017b | + | + |  | + |  |  |  | + |  |  |  |  | + | + | + |  | + | + | + | + |  | + | + |
| Zhang J, 2017 | + | + | + |  |  |  |  | + | + | + |  |  | + |  | + | + |  |  |  |  |  |  | + |
| Gavaskar A, 2018 |  |  |  |  |  |  | + |  |  |  |  |  | + | + |  | + |  | + |  |  |  | + |  |
| Zhang C, 2018 | + | + |  |  |  | + | + | + | + |  |  |  | + | + | + |  |  | + |  |  | + | + |  |
| Imerci A, 2018 |  |  |  |  |  |  | + |  | + |  |  |  | + |  | + | + | + | + |  |  | NE | + |  |
| Duramaz A, 2019 | + | + | + |  |  |  |  | + |  |  | + |  | + |  |  |  |  | + | + |  | + | + |  |
| Ulku T, 2019 | + |  | + |  |  |  | + |  |  |  | + |  | + |  |  | + |  |  |  |  |  |  |  |
| Zhao F, 2021 | + | + |  | + |  | + | + |  | + |  |  |  | + | + |  |  | NE | + | + | + |  |  | + |
| Su Z, 2022 | + | + | + | + |  | + | + |  | + |  |  |  | + |  |  | + |  |  |  | + | + | NE |  |
| Polat G, 2022 |  |  |  |  |  |  |  |  |  |  |  |  | + |  |  |  |  |  |  |  |  | + |  |
| Zhu Z, 2023 | + | + |  | + |  | + | + |  | + |  |  |  | + | + |  |  | + |  |  | + |  |  |  |
| Yalin M, 2023 | + |  | + |  |  |  |  | + |  |  |  |  | + | + |  |  | + | + | + |  | + |  | + |

1. Operative time.

2. Intraoperative blood loss.

3. Fluoroscopy time.

4. Length of hospital stay.

5. HHS at 6 month after surgery.

6. HHS at 1 year after surgery.

7. HHS at final follow up.

8. Time to union/healing.

9. Femoral neck shortening.

10. Time to full bearing.

11. Implant failure.

12. Varus collapse.

13. Femoral shaft fracture.

14. Screw migration.

15. Non-union.

16. Cut-out.

17. Hip and thigh pain.

18. Deep venous thrombosis.

19. Infection.

20. Revision/reoperation.

NE: No events of this outcome were reported in all patients, and the study was excluded from quantitative analysis of this outcome.

Table S3. Risk of bias assessment of randomized controlled trials.

| Study | Random sequence generation | Allocation concealment | Blinding of participants and personnel | Blinding of outcome assessment | Incomplete outcome data | Selective reporting | Other bias |
| --- | --- | --- | --- | --- | --- | --- | --- |
| Zhang S, 2013 | Low risk | Unclear | Unclear | Unclear | Low risk | Low risk | Low risk |
| Seyhan M, 2015 | Unclear | Unclear | Unclear | Unclear | Low risk | Low risk | Low risk |
| Berger-Groch J, 2016 | Unclear | Low risk | Unclear | Low risk | Low risk | Low risk | Low risk |
| Hopp S, 2016 | Unclear | Low risk | Unclear | Unclear | Low risk | Low risk | Low risk |
| Su H, 2016 | Unclear | Unclear | Unclear | Unclear | Low risk | Low risk | Low risk |

Table S4. Quality assessment of observational studies using GRACE checklist.

| Study | Data | | | | | |  | Methods | | | | | Total |
| --- | --- | --- | --- | --- | --- | --- | --- | --- | --- | --- | --- | --- | --- |
|  | D1 | D2 | D3 | D4 | D5 | D6 |  | M1 | M2 | M3 | M4 | M5 |  |
| Wang Y, 2013 | + | + | + | + | + | - |  | + | + | - | - | - | 7 |
| Wu D, 2014 | + | + | + | + | + | + |  | + | + | + | + | - | 10 |
| Yu W, 2016 | + | + | + | + | + | + |  | + | + | + | + | - | 10 |
| Zehir S, 2015 | + | + | + | + | + | + |  | + | + | + | + | - | 10 |
| Zhang H, 2017a | + | + | + | + | + | + |  | + | + | - | + | - | 9 |
| Serrano R, 2017 | + | + | + | + | + | + |  | + | + | - | + | - | 9 |
| Zhang H, 2017b | + | + | + | + | + | + |  | + | + | + | + | - | 10 |
| Zhang J, 2017 | + | + | + | + | + | + |  | + | + | + | + | - | 10 |
| Gavaskar A, 2018 | + | + | + | + | + | + |  | + | + | + | + | - | 10 |
| Zhang C, 2018 | + | + | + | + | + | + |  | + | + | + | + | - | 10 |
| Imerci A, 2018 | + | + | + | + | + | + |  | + | + | + | + | - | 10 |
| Duramaz A, 2019 | + | + | + | + | + | + |  | + | + | - | + | - | 9 |
| Ulku T, 2019 | + | + | + | + | + | + |  | + | + | - | + | - | 9 |
| Zhao F, 2021 | + | + | + | + | + | + |  | + | + | + | + | - | 10 |
| Su Z, 2022 | + | + | + | + | + | + |  | + | + | + | + | - | 10 |
| Polat G, 2022 | + | + | + | + | + | + |  | + | + | - | - | - | 8 |
| Zhu Z, 2023 | + | + | + | + | + | + |  | + | + | + | + | - | 10 |
| Yalin M, 2023 | + | + | + | + | + | - |  | + | + | - | + | - | 8 |

GRACE: Good Research for Comparative Effectiveness.

D1: Were treatment and/or important details of treatment exposure adequately recorded for the study purpose in the data source(s)?

D2: Were the primary outcomes adequately recorded for the study purpose (e.g., available in sufficient detail through data source(s))?

D3: Was the primary clinical outcome(s) measured objectively rather than subject to clinical judgment (e.g., opinion about whether the patient's condition has improved)?

D4: Were primary outcomes validated, adjudicated, or otherwise known to be valid in a similar population?

D5: Was the primary outcome(s) measured or identified in an equivalent manner between the treatment/ intervention group and the comparison group(s)?

D6: Were important covariates that may be known confounders or effect modifiers available and recorded?

M1: Was the study (or analysis) population restricted to new initiators of treatment or those starting a new course of treatment?

M2: If one or more comparison groups were used, were they concurrent comparators? If not, did the authors justify the use of historical comparisons group(s)?

M3: Were important covariates, confounding and effect modifying variables taken into account in the design and/or analysis?

M4: Is the classification of exposed and unexposed person-time free of "immortal time bias"?

M5: Were any meaningful analyses conducted to test key assumptions on which primary results are based?

Table S5. Results of subgroup analyses.

| Outcomes | No. of studies | Sample size | Heterogeneity | | Model | Pooled results | | |
| --- | --- | --- | --- | --- | --- | --- | --- | --- |
|  |  |  | I^2^ (%) | P |  | Estimates | 95%CI | P |
| Operative time |  |  |  |  |  | MD (minutes) |  |  |
| Gamma3 | 5 | 310/398 | 97.9 | <0.001 | RE | 4.55 | -10.08 to 19.19 | 0.542 |
| PFNA/PFNA-II | 13 | 923/971 | 99.1 | <0.001 | RE | 6.44 | -0.13 to 13.02 | 0.055 |
| RCT | 5 | 233/237 | 93.6 | <0.001 | RE | 1.51 | -9.87 to 12.89 | 0.795 |
| Observational study | 13 | 1000/1132 | 99.2 | <0.001 | RE | 7.50 | 0.75 to 14.25 | 0.030 |
| Unstable type only | 12 | 930/1018 | 98.6 | <0.001 | RE | 9.19 | 2.91 to 15.47 | 0.004 |
| Mixed types | 6 | 303/351 | 98.8 | <0.001 | RE | -0.72 | -12.06 to 10.62 | 0.901 |
| Intraoperative blood loss |  |  |  |  |  | MD (mL) |  |  |
| Gamma3 | 4 | 255/349 | 99.1 | <0.001 | RE | -0.79 | -38.92 to 37.35 | 0.968 |
| PFNA/PFNA-II | 10 | 781/805 | 97.0 | <0.001 | RE | 24.51 | 13.07 to 35.95 | <0.001 |
| RCT | 3 | 146/145 | 88.5 | <0.001 | RE | -8.97 | -74.55 to 56.61 | 0.789 |
| Observational study | 11 | 890/1009 | 98.0 | <0.001 | RE | 23.80 | 14.24 to 33.35 | <0.001 |
| Unstable type only | 12 | 930/1018 | 98.4 | <0.001 | RE | 21.06 | 8.02 to 34.10 | 0.002 |
| Mixed types | 2 | 106/136 | 0 | 0.389 | FE | 4.56 | 1.74 to 7.39 | 0.002 |
| Fluoroscopy time |  |  |  |  |  | MD (minutes) |  |  |
| Gamma3 | 1 | 87/174 | - | - | - | - | - | - |
| PFNA/PFNA-II | 9 | 552/588 | 99.8 | <0.001 | RE | 0.72 | 0.32 to 1.13 | <0.001 |
| RCT | 2 | 89/99 | 99.9 | <0.001 | RE | 0.74 | -0.75 to 2.23 | 0.330 |
| Observational study | 8 | 550/663 | 99.8 | <0.001 | RE | 0.66 | 0.33 to 0.99 | <0.001 |
| Unstable type only | 6 | 411/496 | 99.8 | <0.001 | RE | 1.16 | 0.59 to 1.74 | <0.001 |
| Mixed types | 4 | 228/266 | 99.2 | <0.001 | RE | -0.05 | -0.27 to 0.18 | 0.676 |
| HHS (1 year) |  |  |  |  |  | MD |  |  |
| Gamma3 | 3 | 216/310 | 34.0 | 0.220 | FE | 0.67 | -0.27 to 1.60 | 0.161 |
| PFNA/PFNA-II | 8 | 435/430 | 21.5 | 0.259 | FE | 0.95 | 0.11 to 1.78 | 0.026 |
| RCT | 2 | 82/93 | 64.6 | 0.093 | RE | 0.27 | -2.55 to 3.08 | 0.853 |
| Observational study | 9 | 569/647 | 13.6 | 0.321 | FE | 0.85 | 0.17 to 1.54 | 0.014 |
| Unstable type only | 9 | 607/681 | 16.6 | 0.295 | FE | 0.64 | -0.04 to 1.31 | 0.066 |
| Mixed types | 2 | 44/59 | 0 | 0.431 | FE | 1.86 | 0.27 to 3.45 | 0.022 |
| Time to union/healing |  |  |  |  |  | MD (weeks) |  |  |
| Gamma3 | 2 | 129/136 | 0 | 0.415 | FE | -0.26 | -0.92 to 0.40 | 0.436 |
| PFNA/PFNA-II | 10 | 605/630 | 71.9 | <0.001 | RE | -0.77 | -1.36 to -0.19 | 0.010 |
| RCT | 3 | 139/149 | 80.1 | 0.007 | RE | -0.81 | -2.77 to 1.41 | 0.415 |
| Observational study | 9 | 595/617 | 66.5 | 0.002 | RE | -0.64 | -1.15 to -0.14 | 0.012 |
| Unstable type only | 10 | 682/687 | 72.2 | 0.002 | RE | -0.77 | -1.34 to -0.19 | 0.009 |
| Mixed types | 2 | 52/79 | 0 | 0.626 | FE | -0.16 | -0.96 to 0.63 | 0.688 |
| Implant failures |  |  |  |  |  | RR |  |  |
| Gamma3 | 5 | 538/479 | 56.2 | 0.058 | RE | 0.43 | 0.19 to 1.01 | 0.052 |
| PFNA/PFNA-II | 15 | 1177/1118 | 30.9 | 0.122 | FE | 0.30 | 0.23 to 0.40 | <0.001 |
| RCT | 3 | 136/135 | 59.5 | 0.085 | RE | 0.46 | 0.11 to 2.00 | 0.302 |
| Observational study | 17 | 1579/1462 | 36.2 | 0.068 | RE | 0.36 | 0.26 to 0.50 | <0.001 |
| Unstable type only | 14 | 1006/1091 | 34.5 | 0.100 | FE | 0.28 | 0.21 to 0.38 | <0.001 |
| Mixed types | 6 | 709/506 | 31.4 | 0.200 | FE | 0.48 | 0.31 to 0.73 | <0.001 |
| Cut-out |  |  |  |  |  | RR |  |  |
| Gamma3 | 4 | 496/435 | 27.3 | 0.248 | FE | 0.28 | 0.13 to 0.62 | 0.002 |
| PFNA/PFNA-II | 10 | 886/896 | 15.3 | 0.303 | FE | 0.31 | 0.18 to 0.54 | <0.001 |
| RCT | 2 | 94/91 | 0 | 0.991 | FE | 0.19 | 0.03 to 1.08 | 0.062 |
| Observational study | 12 | 1288/1240 | 23.3 | 0.215 | FE | 0.31 | 0.20 to 0.50 | <0.001 |
| Unstable type only | 10 | 829/906 | 0 | 0.655 | FE | 0.23 | 0.13 to 0.40 | <0.001 |
| Mixed types | 4 | 553/425 | 40.1 | 0.171 | FE | 0.61 | 0.29 to 1.29 | 0.198 |
| Hip and thigh pain |  |  |  |  |  | RR |  |  |
| Gamma3 | 3 | 213/305 | 0 | 0.832 | FE | 0.83 | 0.46 to 1.52 | 0.549 |
| PFNA/PFNA-II | 7 | 584/603 | 45.6 | 0.088 | RE | 0.63 | 0.41 to 0.96 | 0.032 |
| RCT | 3 | 126/134 | 0 | 0.526 | FE | 0.47 | 0.23 to 0.99 | 0.046 |
| Observational study | 7 | 671/774 | 32.8 | 0.178 | FE | 0.74 | 0.57 to 0.97 | 0.032 |
| Unstable type only | 7 | 581/658 | 0 | 0.639 | FE | 0.51 | 0.36 to 0.74 | <0.001 |
| Mixed types | 3 | 216/250 | 0 | 0.623 | FE | 1.01 | 0.71 to 1.48 | 0.946 |
| Revision/reoperation |  |  |  |  |  | RR |  |  |
| Gamma3 | 3 | 173/258 | 11.2 | 0.324 | FE | 0.60 | 0.21 to 1.68 | 0.328 |
| PFNA/PFNA-II | 8 | 771/694 | 11.0 | 0.345 | FE | 0.36 | 0.23 to 0.54 | <0.001 |
| RCT | 3 | 133/130 | 7.2 | 0.340 | FE | 0.54 | 0.19 to 1.58 | 0.261 |
| Observational study | 8 | 811/822 | 15.7 | 0.306 | FE | 0.36 | 0.24 to 0.55 | <0.001 |
| Unstable type only | 9 | 714/787 | 0 | 0.550 | FE | 0.33 | 0.22 to 0.51 | <0.001 |
| Mixed types | 2 | 230/165 | 0 | 0.479 | FE | 1.12 | 0.37 to 3.37 | 0.847 |

FE: fixed-effect model; MD: mean difference; PFNA: proximal femoral nail anti-rotation; RCT: randomized controlled trial; RE: random-effect model; RR: risk ratio.
