# Supplementary material for: Dual-screw versus single-screw cephalomedullary nails for intertrochanteric femoral fractures: a systematic review and meta-analysis
Source: J Orthop Surg Res. 2023 Aug 20;18:607. doi: 10.1186/s13018-023-04103-x (PMC10440877; doi:10.1186/s13018-023-04103-x)
Supplement: Supplementary file 4 — Additional file 4. Figure S1 Forest plot of meta-analysis of operative time. Figure S2 Forest plot of meta-analysis of fluoroscopy time. Figure S3 Forest plot of meta-analysis of intraoperative blood loss. Figure S4 Forest plot of meta-analysis of length of hospital stay. Figure S5 Forest plot of meta-analysis of femoral neck shortening. Figure S6 Forest plot of meta-analysis of time to full bearing. Figure S7 Forest plot of meta-analysis of 6-month Harris Hip Score. Figure S8 Forest plot of meta-analysis of Harris Hip Score at last follow-up. Figure S9 Forest plot of meta-analysis of femoral shaft fracture. Figure S10 Forest plot of meta-analysis of cut-out. Figure S11 Forest plot of meta-analysis of screw migration. Figure S12 Forest plot of meta-analysis of varus collapse. Figure S13 Forest plot of meta-analysis of non-union. Figure S14 Forest plot of meta-analysis of infection. Figure S15 Forest plot of meta-analysis of deep venous thrombosis. Figure S16 Forest plot of meta-analysis of mortality. Figure S17 Sensitivity analysis of operative time using the “Leave-one-out” method. [file 13018_2023_4103_MOESM4_ESM.docx]

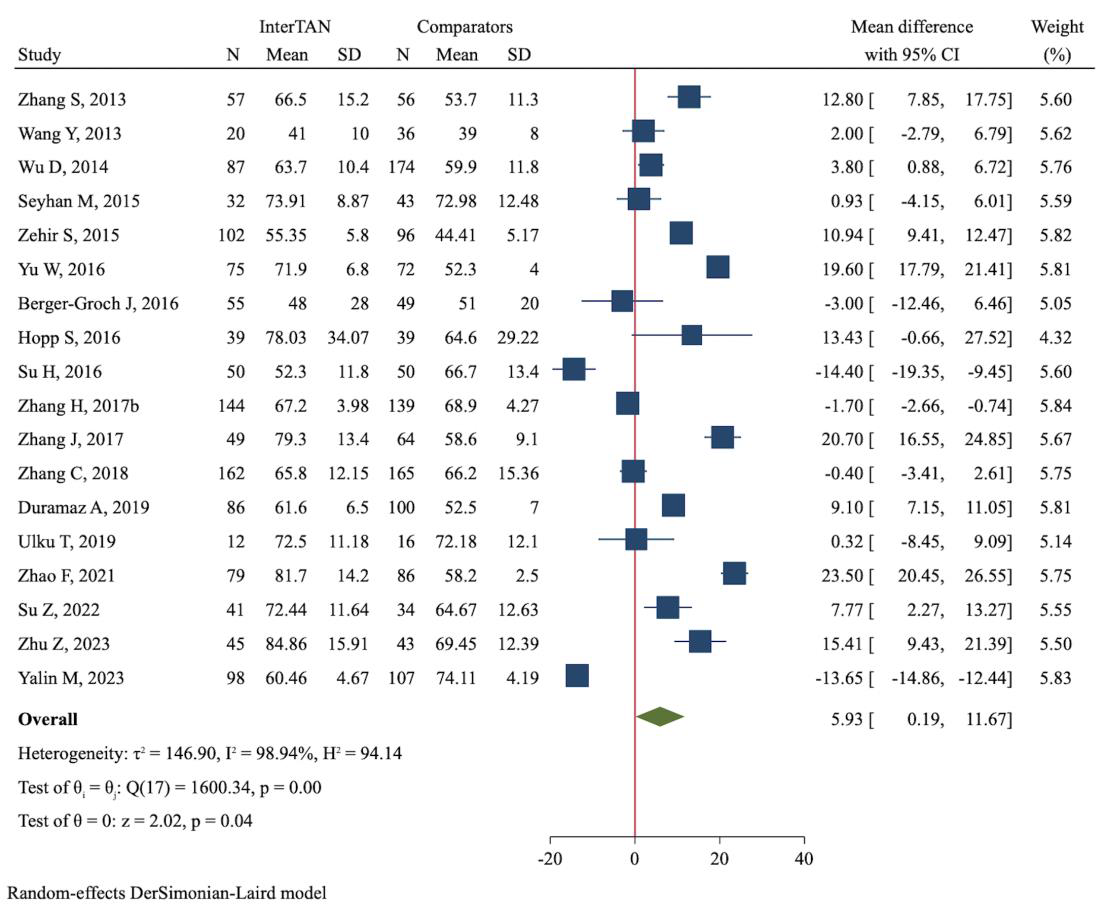


Figure S1 Forest plot of meta-analysis of operative time


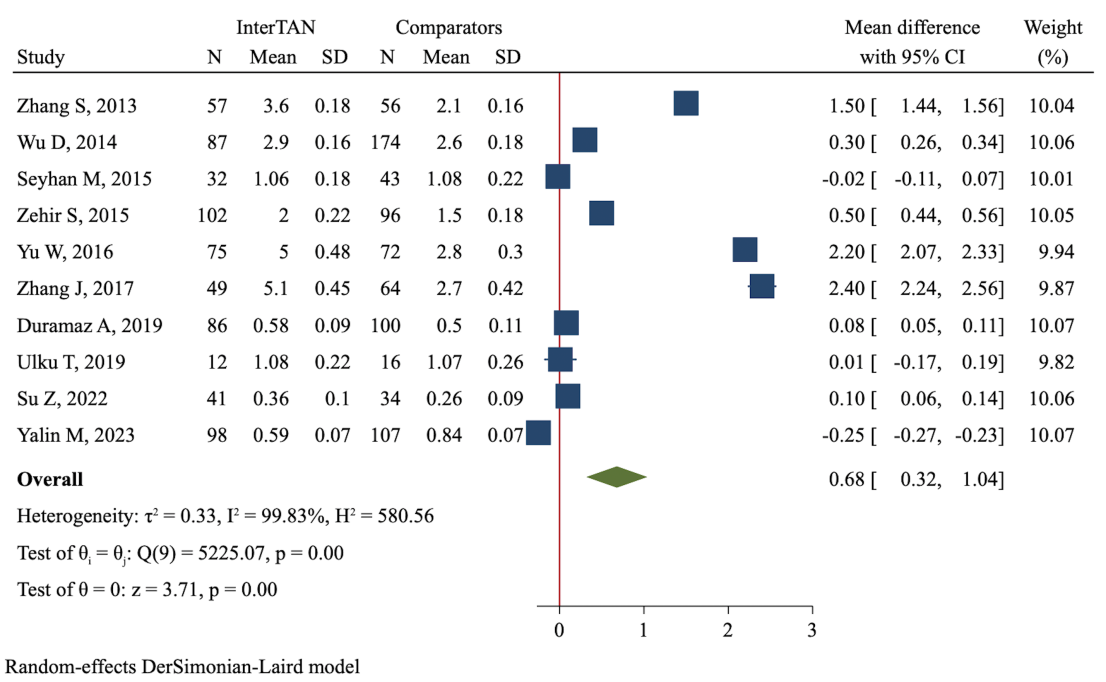


Figure S2 Forest plot of meta-analysis of fluoroscopy time


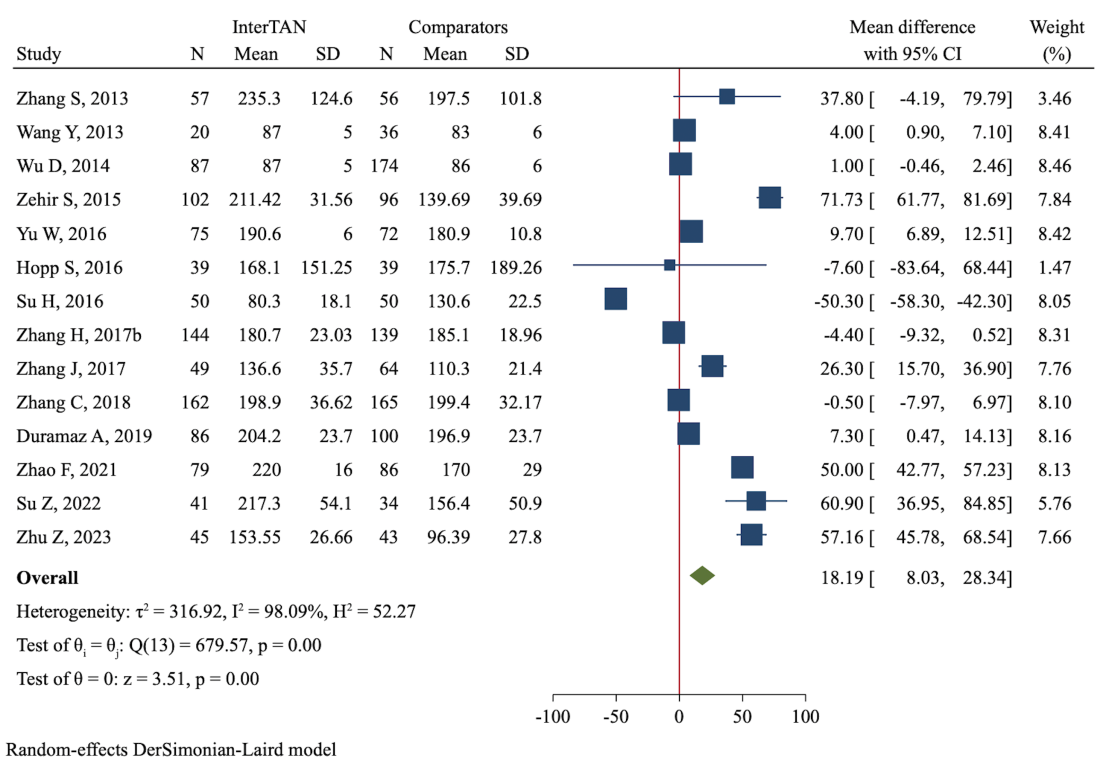


Figure S3 Forest plot of meta-analysis of intraoperative blood loss


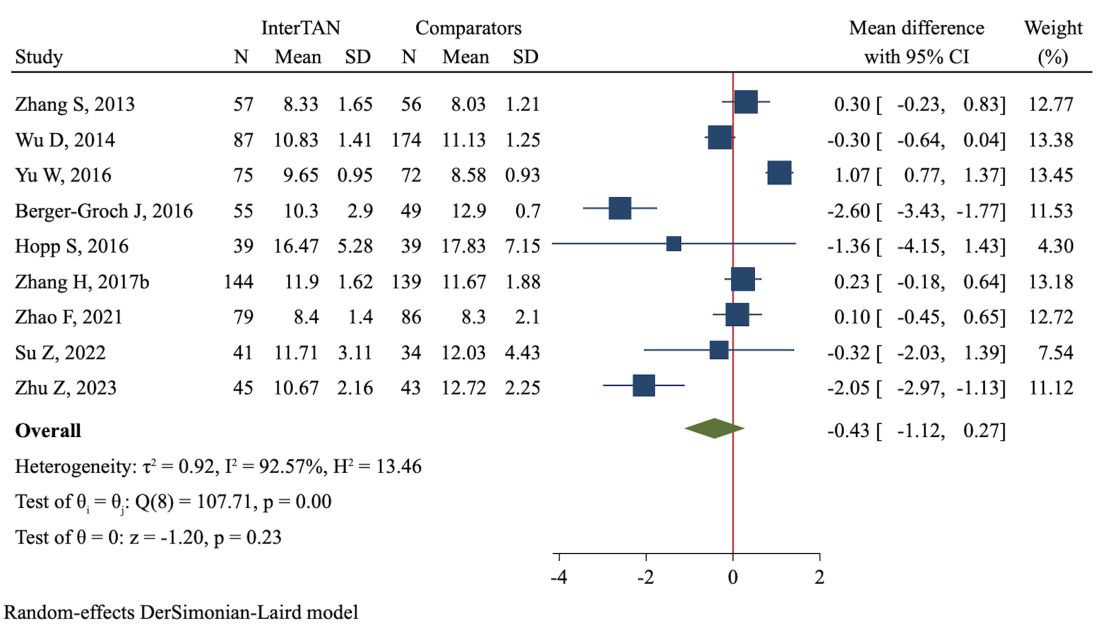


Figure S4 Forest plot of meta-analysis of length of hospital stay


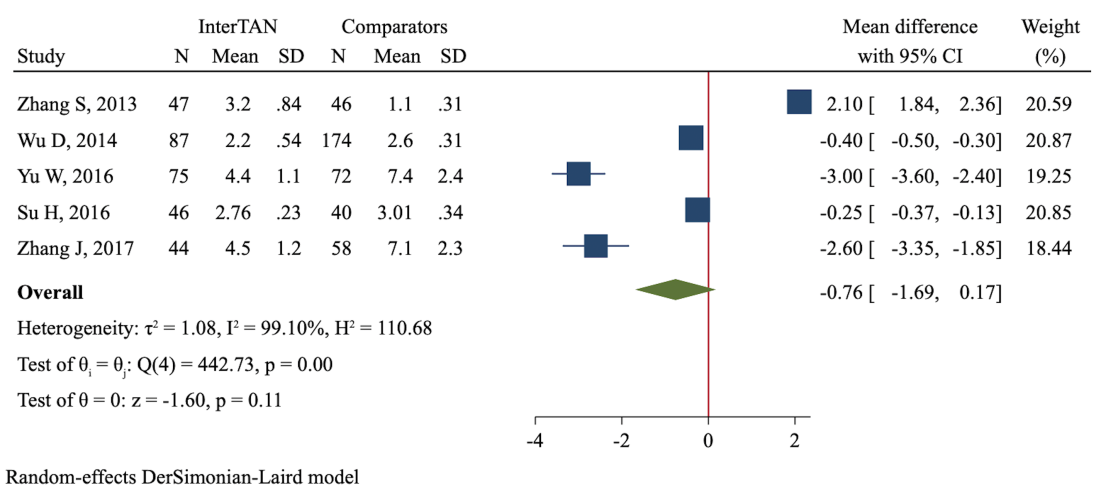


Figure S5 Forest plot of meta-analysis of femoral neck shortening


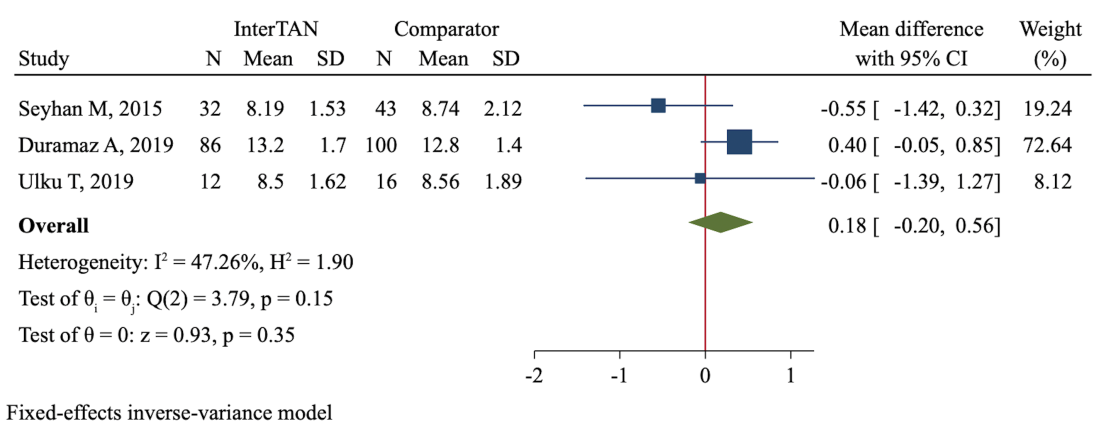


Figure S6 Forest plot of meta-analysis of time to full bearing


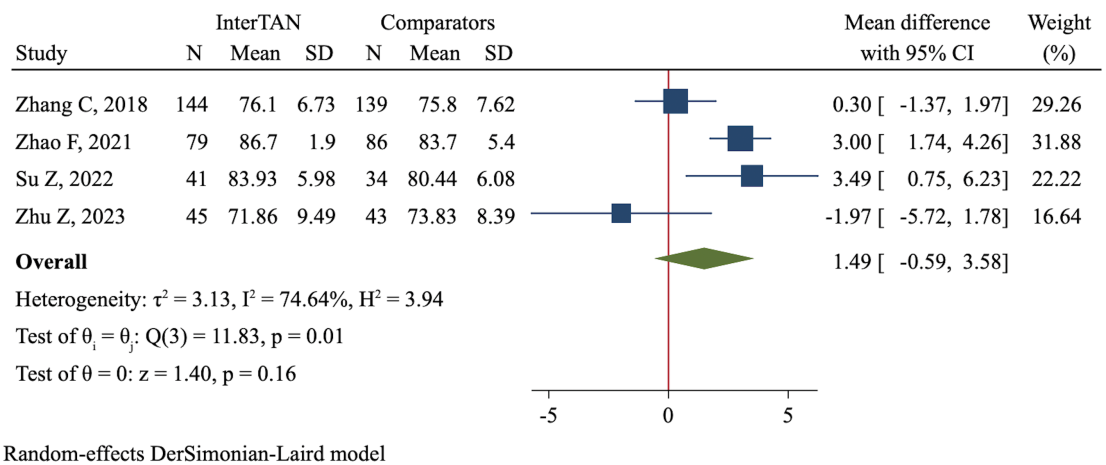


Figure S7 Forest plot of meta-analysis of 6-month Harris Hip Score


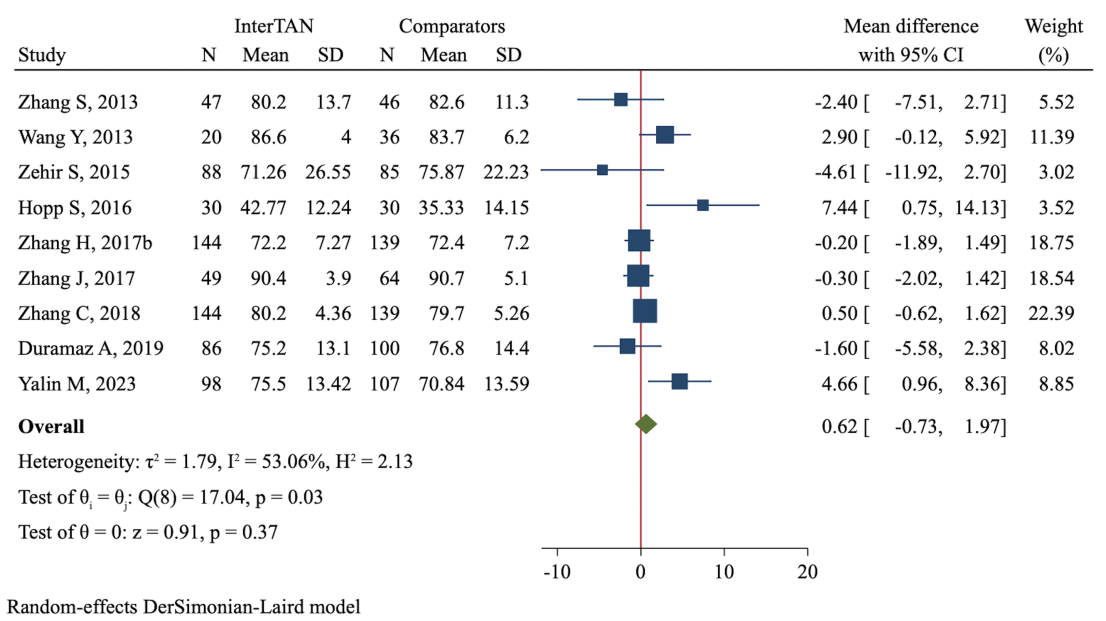


Figure S8 Forest plot of meta-analysis of Harris Hip Score at last follow up


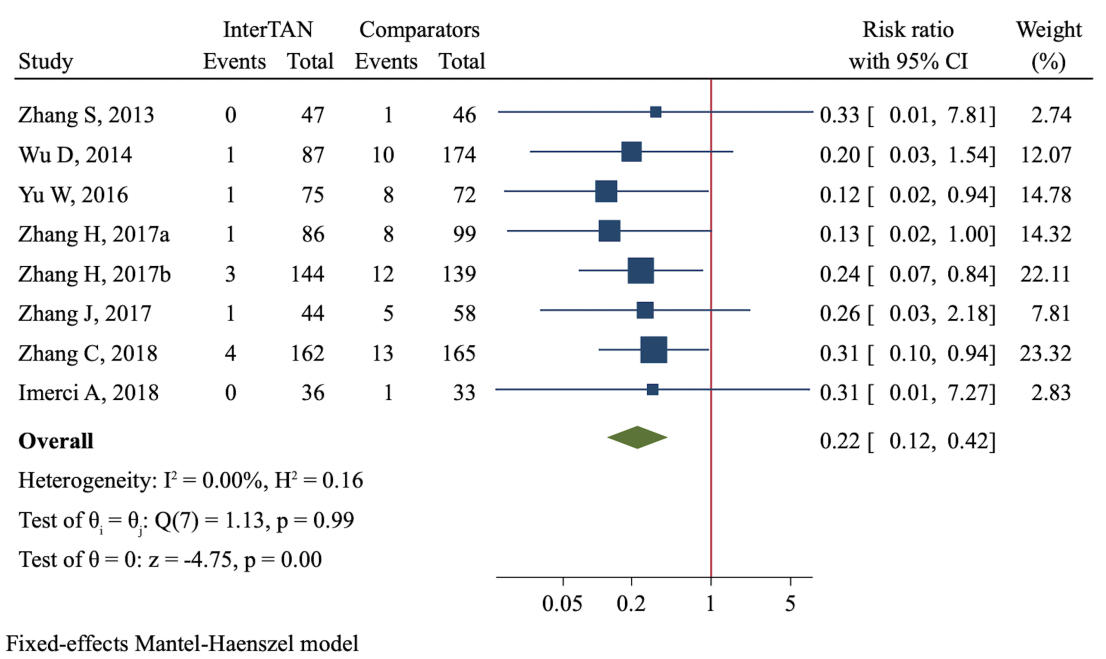


Figure S9 Forest plot of meta-analysis of femoral shaft fracture


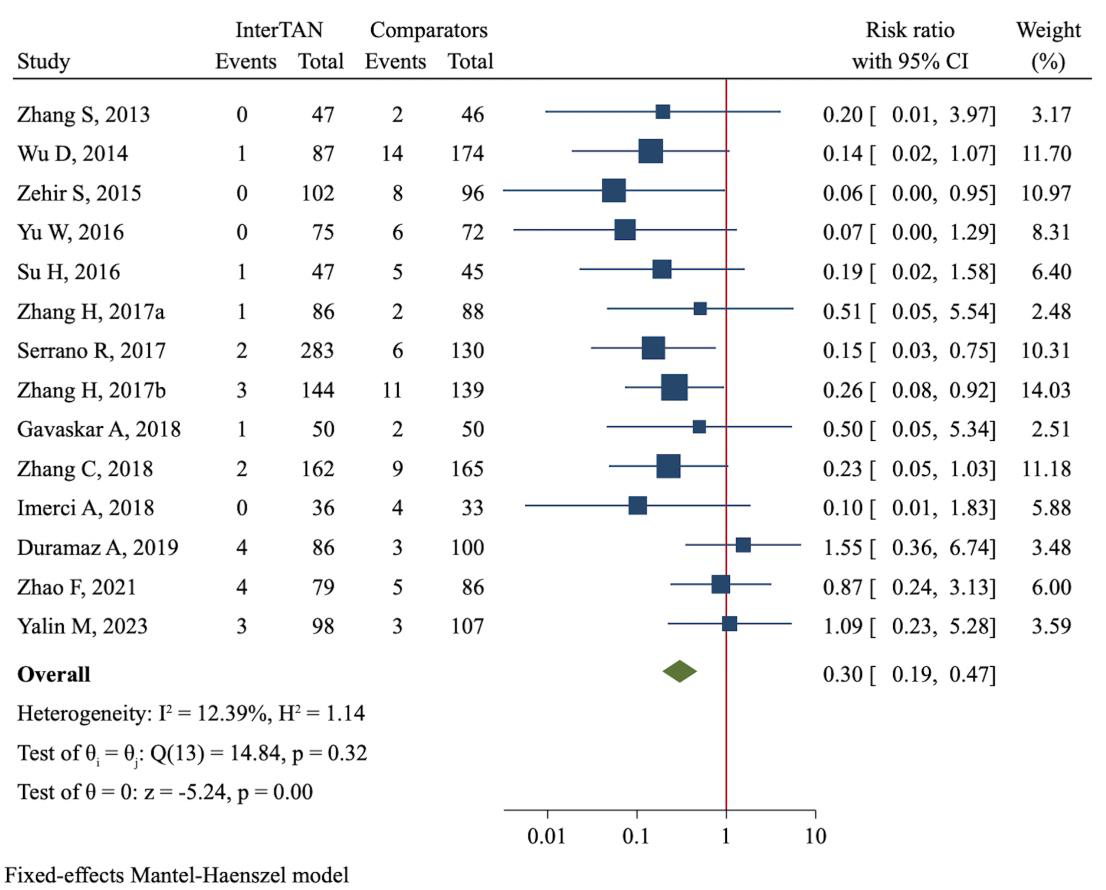


Figure S10 Forest plot of meta-analysis of cut-out


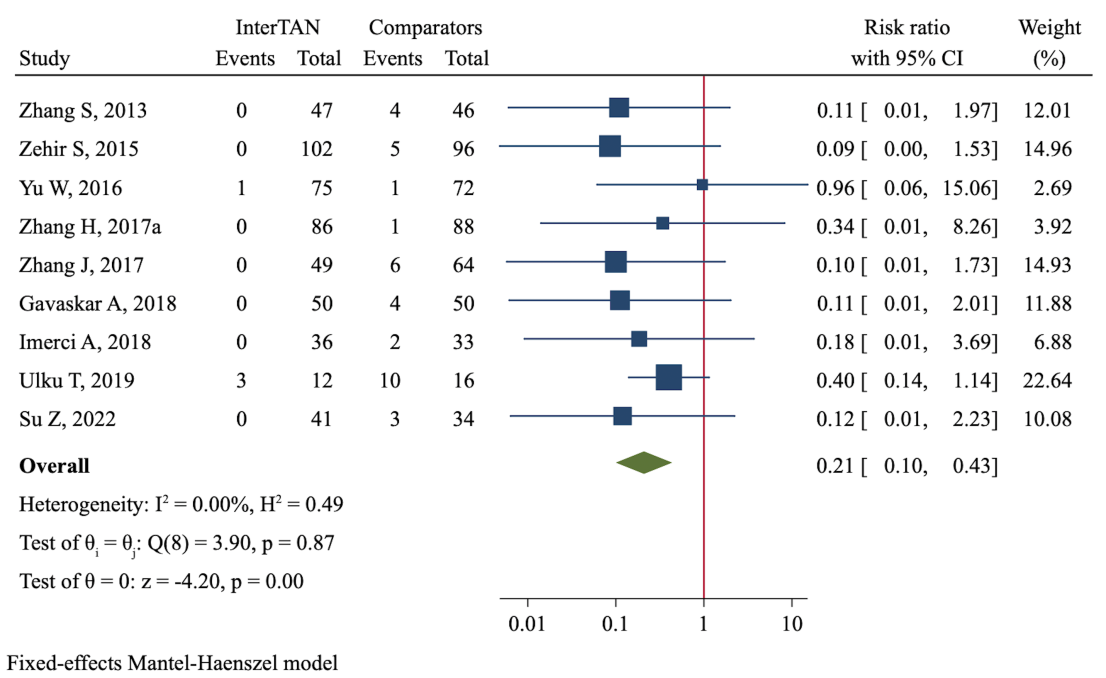


Figure S11 Forest plot of meta-analysis of screw migration


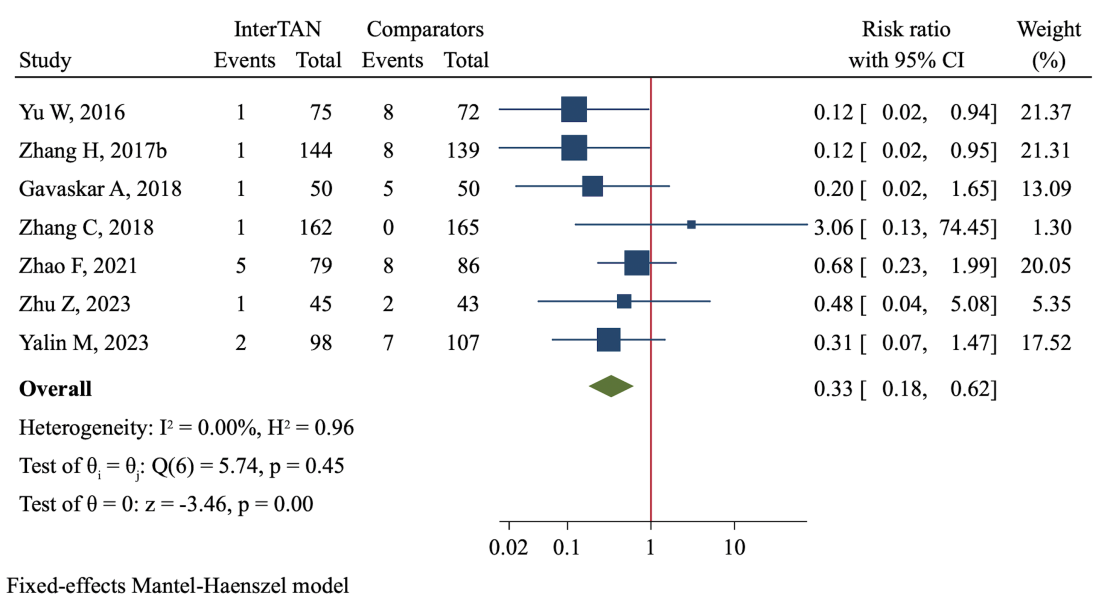


Figure S12 Forest plot of meta-analysis of varus collapse


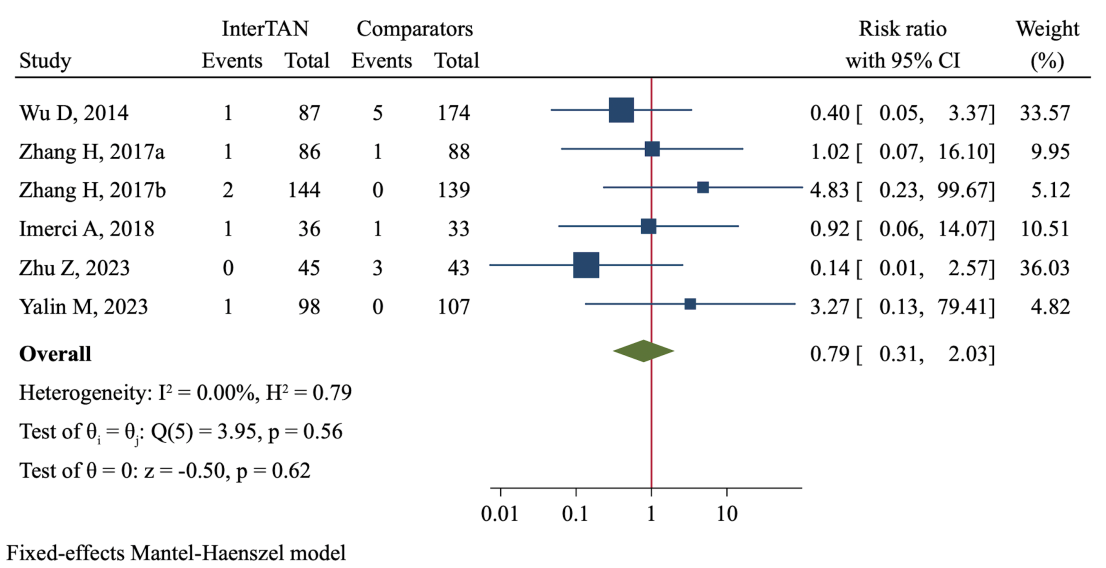


Figure S13 Forest plot of meta-analysis of non-union


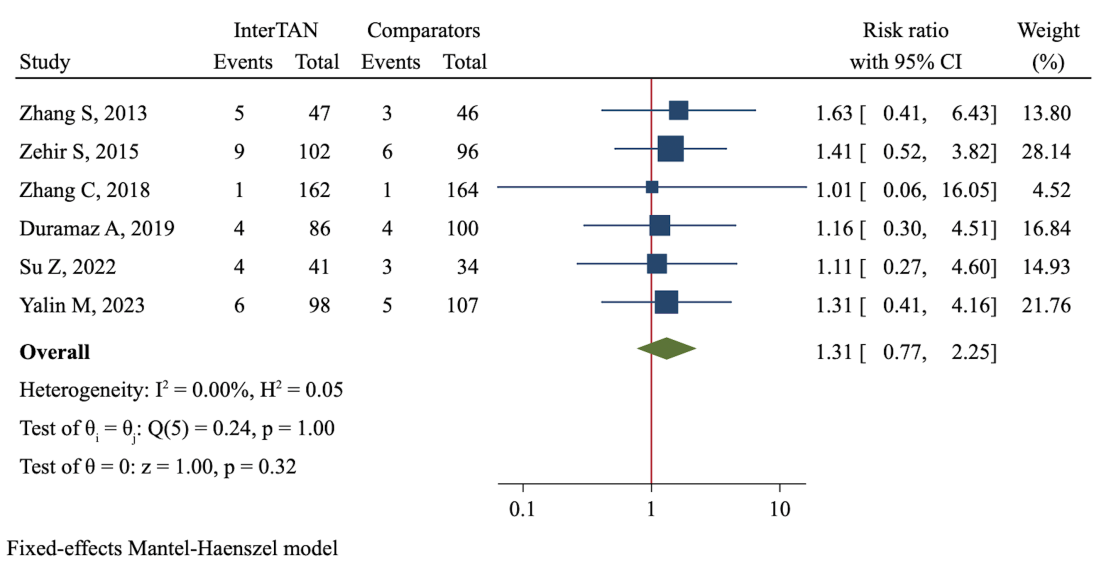


Figure S14 Forest plot of meta-analysis of infection


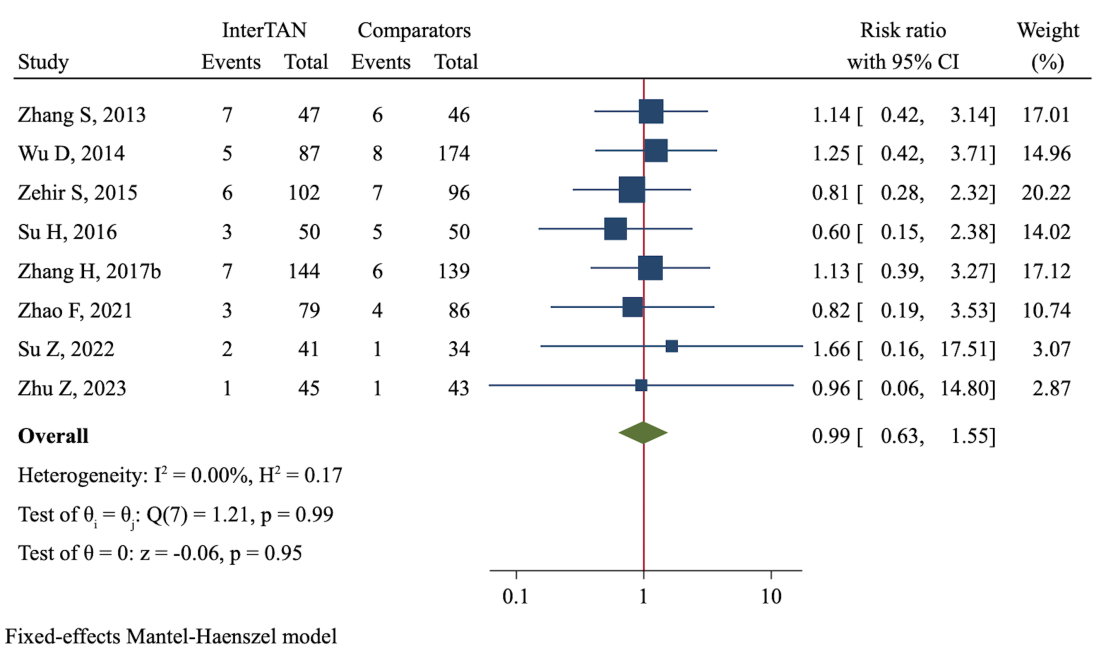


Figure S15 Forest plot of meta-analysis of deep venous thrombosis


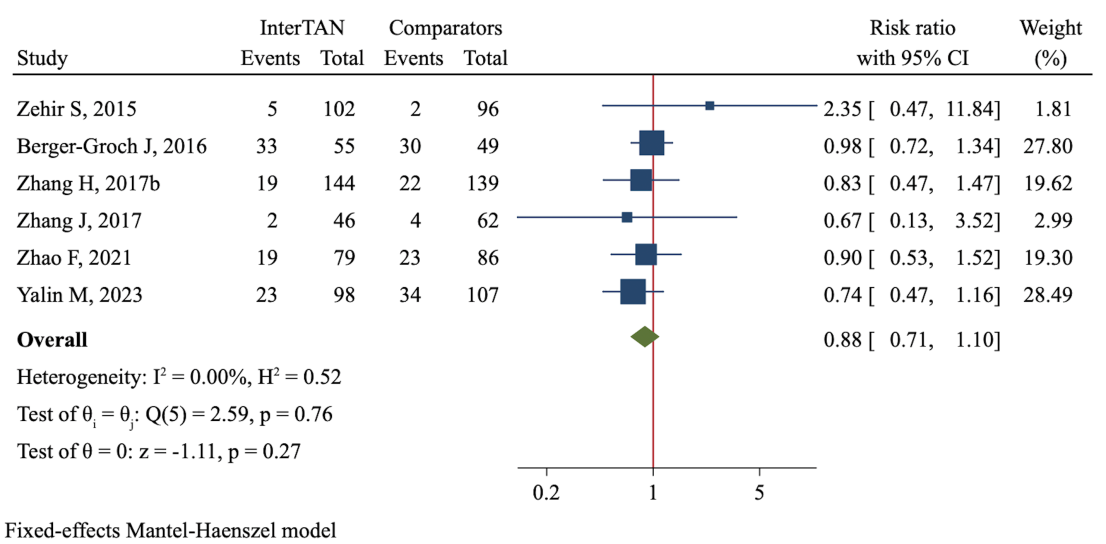


Figure S16 Forest plot of meta-analysis of mortality


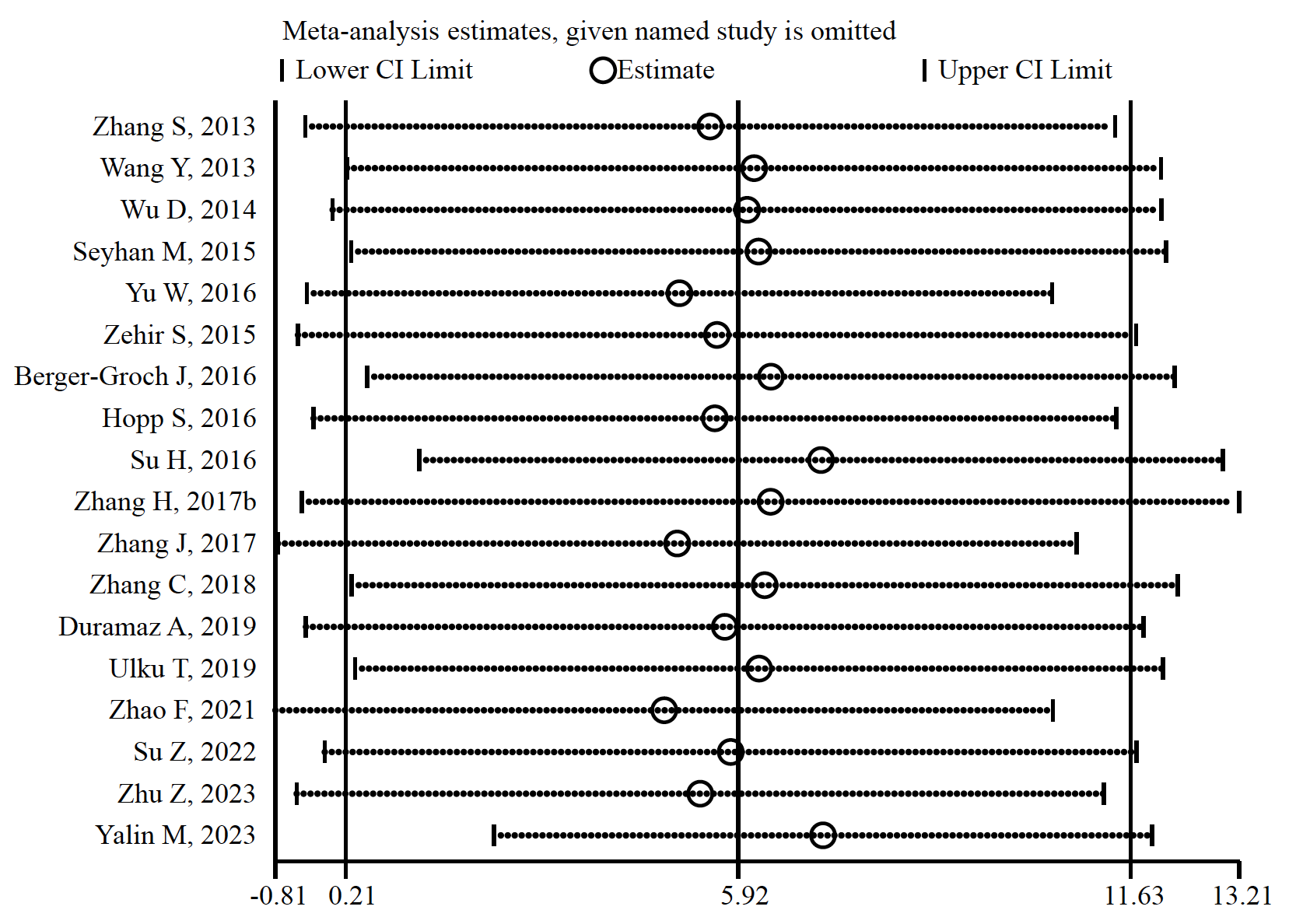


Figure S17 Sensitivity analysis of operative time using “Leave-one-out” method
